# Defective neurogenesis and schizophrenia-like behavior in PARP-1-deficient mice

**DOI:** 10.1038/s41419-019-2174-0

**Published:** 2019-12-09

**Authors:** Seokheon Hong, Jee Hyun Yi, Soonje Lee, Chang-Hwan Park, Jong Hoon Ryu, Ki Soon Shin, Shin Jung Kang

**Affiliations:** 1 Department of Molecular Biology, Sejong University, Seoul, 05006 Republic of Korea; 2 Department of Biology, Kyung Hee University, Seoul, 02447 Republic of Korea; 3 Department of Microbiology, College of Medicine, Hanyang University, Seoul, 04763 Republic of Korea; 4 Department of Life and Nanopharmaceutical Sciences, Kyung Hee University, Seoul, 02447 Republic of Korea; 5 Department of Integrative Bioscience and Biotechnology, Sejong University, Seoul, 05006 Republic of Korea; 6 Present Address: Center for Biomaterials, Biomedical Research Institute, Korea Institute of Science and Technology, Seoul, 02792 Republic of Korea; 7 Present Address: Center for Synaptic Brain Dysfunction, Institute for Basic Science, Daejeon, 34126 Republic of Korea

**Keywords:** Neural progenitors, Neural stem cells

## Abstract

In the current study we present evidence suggesting that PARP-1 regulates neurogenesis and its deficiency may result in schizophrenia-like behavioral deficits in mice. PARP-1 knockout neural stem cells exhibited a marked upregulation of embryonic stem cell phosphatase that can suppress the proliferative signaling of PI3K-Akt and ERK. The suppressed activity of Akt and ERK in the absence of PARP-1 results in the elevation of FOXO1 activity and its downstream target genes p21 and p27, leading to the inhibition of neural stem cell proliferation. Moreover, expression of neurogenic factors and neuronal differentiation were decreased in the PARP-1 knockout neural stem cells whereas glial differentiation was increased. In accordance with the in vitro data, PARP-1 knockout mice exhibited reduced brain weight with enlarged ventricle as well as decreased adult neurogenesis in the hippocampus. Interestingly, PARP-1 knockout mice exhibited schizophrenia-like symptoms such as anxiety, depression, social interaction deficits, cognitive impairments, and prepulse inhibition deficits. Taken together, our results suggest that PARP-1 regulates neurogenesis during development and in adult and its absence may lead to the schizophrenia-like behavioral abnormality in mice.

## Introduction

Poly(ADP-ribose) polymerase-1 (PARP-1) is a multifunctional nuclear enzyme that regulates DNA repair, gene expression and cell death by attaching poly(ADP-ribose) (PAR) on many important regulators of these processes^1^. When overactivated by massive DNA damage, PARP-1 can induce cell death by depleting NAD^+^^2^. However, PARP-1 can act as a survival factor under tolerable DNA damage condition by detecting and repairing the damage^3^. PARP-1-deficient cells exhibit higher rate of genomic instability and enhanced susceptibility toward genotoxic insults^1^. Thus PARP-1 has been extensively studied as a target for cancer pharmacotherapy^4^. Furthermore, there have been studies suggesting that PARP-1 is involved in the regulation of cell proliferation. Mouse embryonic fibroblasts from the PARP-1 knockout (KO) mice exhibited decreased proliferation^5^. Another study suggested that PARP-1 can regulate cell proliferation by directly repressing FOXO1^6^. There has been a report showing that inhibitors of PARP-1 attenuated the activation of a prosurvival kinase Akt^7^. These studies suggest a possible role of PARP-1 as a pro-proliferative factor.

It has been suggested that a subtype of schizophrenia is a neurodevelopmental disorder^8,9^. Defective neurogenesis has been reported in animal models and patients of schizophrenia^9–12^. Specifically, reduction in NSC proliferation, decreased cerebral and hippocampal volume, enlarged ventricle and evidence of defective neuronal migration have been reported^9,10,13^. Supporting the neurodevelopmental disorder hypothesis of schizophrenia, a number of susceptibility genes have been identified and many of them are involved in different aspects of neurogenesis^14–17^. Disrupted-in-schizophrenia 1 (DISC1), one of the schizophrenia susceptibility gene, has been shown to regulate NSC proliferation by regulating Akt-glycogen synthase kinase (GSK) 3β signaling^11,12^.

Akt1, one of the Akt isoforms, has been independently identified as one of the schizophrenia-associated genes^14,18^. Various neuronal signaling pathways induced by growth factors or neurotransmitters like dopamine converge on Akt to regulate cell survival, proliferation, and migration^19^. In schizophrenic patients as well as in animal models, Akt level or activity has been shown to be decreased^19–21^. Consistent with these reports, Akt interacts with many schizophrenia susceptibility gene products such as DISC1, neuregulin1, and dysbindin1^19^. In addition, many downstream targets of Akt including GSK3, FOXO1, β-catenin, and mammalian target of rapamycin have been also implicated in the pathogenesis of schizophrenia and other related mental disorders^19,22^.

In addition to the possible involvement in the regulation of cell proliferation, PARP-1 has been shown to play a role in embryonic stem cell pluripotency and reprogramming^23–25^. An earlier study reported that PARP-1 is required for the HES1 function in neural stem cells^26^. These findings prompted us to hypothesize that PARP-1 may play a role in neural stem cell proliferation and differentiation and its absence may be relevant to the development of psychiatric disorders.

In the present study, we present evidence suggesting that PARP-1 promotes neurogenesis by regulating PI3K, Akt, ERK, and FOXO1 signaling *via* PARP activity-dependent regulation of embryonic stem cell phosphatase (ESP) expression. PARP-1-deficient mice exhibited reduced brain weight and schizophrenia-related behavioral deficits. Our study suggests PARP-1 as a regulator of neurogenesis and a candidate gene associated with schizophrenia-related mental disorders.

## Results

### PARP-1-deficient NSCs exhibit defects in proliferation

**Fig. 1:**
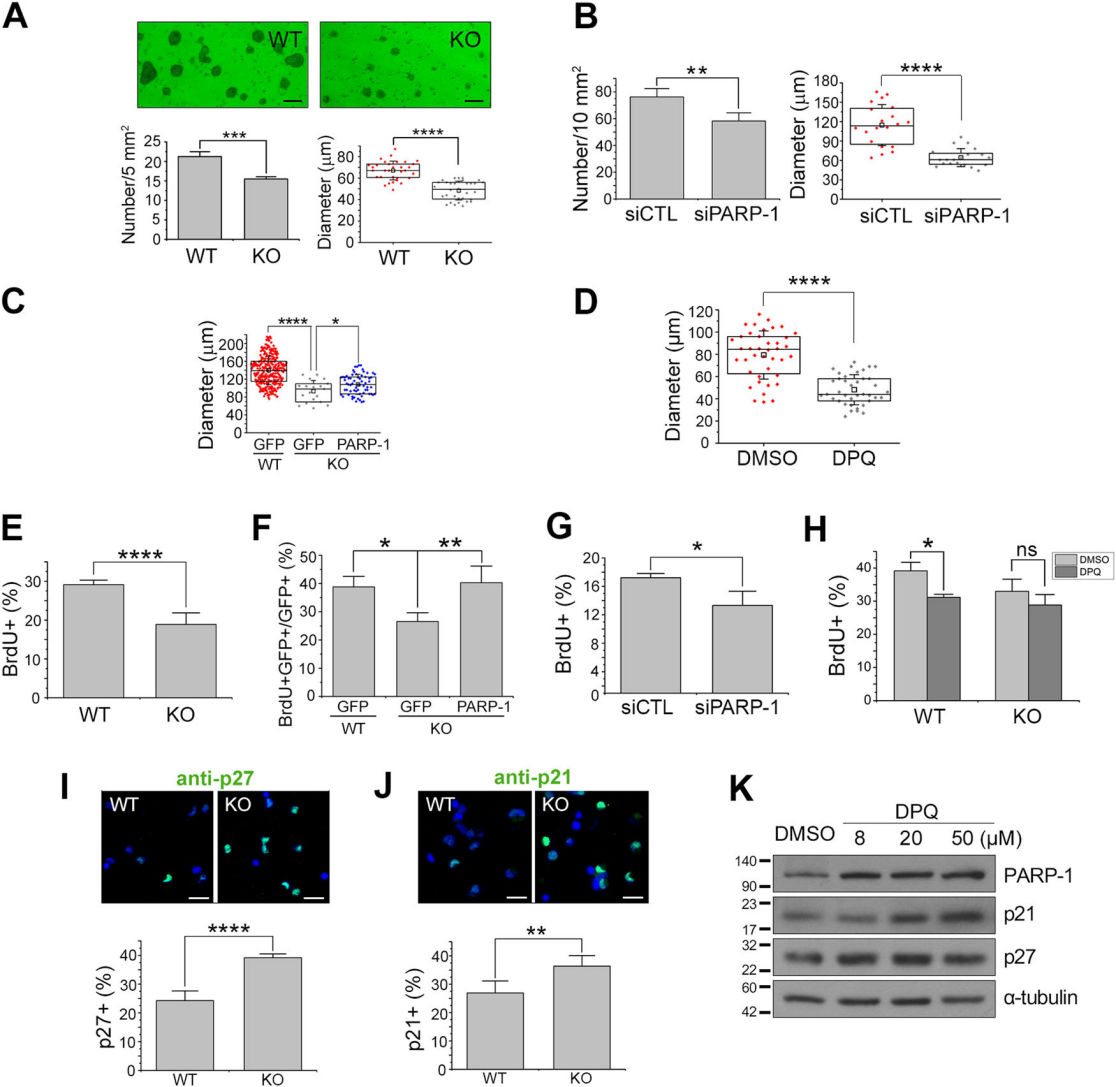
PARP-1-deficient embryonic NSCs exhibited defects in proliferation. **a** NSCs were cultured from E13.5 PARP-1 littermate embryos and sphere formation assay was performed. Representative photographs are shown in the top panels (scale bar = 100 μm). The number (bottom left panel; *n* = 4 independent cultures) and diameter (bottom right; *n* = 32; pooled from four independent cultures) of the primary neurospheres were determined at days in vitro 3. **b** Embryonic NSCs were transfected with PARP-1 siRNA and then assayed for neurosphere formation at day 6 (left panel, *n* = 4 independent cultures; right panel, *n* = 20; pooled from four independent cultures). **c** NSCs prepared from WT or PARP-1 KOs were infected with retroviruses carrying GFP or GFP-PARP-1 (PARP-1) and then assayed for neurosphere formation at day 4 (WT GFP, *n* = 289; KO GFP, *n* = 19; KO PARP-1, *n* = 66; data pooled from four independent cultures). **d** Neurospheres were formed in the presence of a PARP-1 inhibitor DPQ (50 μM) for 5 days (DMSO, *n* = 40; DPQ, *n* = 39, data pooled from four independent cultures). **e**–**f** PARP-1 littermate embryonic NSCs (**e**) or NSCs infected with retroviruses as indicated (**f**) were labeled with BrdU and then quantified (*n* = 6 for each group). **g** PARP-1 siRNA-transfected NSCs were labeled with BrdU and then counted (*n* = 4 for each group). **h** PARP-1 NSCs were incubated with DPQ and then BrdU-labeled (*n* = 44 for each group, pooled from four independent cultures). **i**–**j** PARP-1 NSCs were immunostained for p27 (I; *n* = 8 for each group) and p21 (J; WT, *n* = 4; KO, *n* = 5) and then the immunoreactive cells were counted. Representative images are shown in the upper panels (scale bar = 20 μm). **k** WT NSCs were incubated with increasing concentrations of DPQ (8, 20, 50 μM) and then immunoblotted to monitor the changes in the protein amounts of p21 and p27. Molecular weights on the left in kDa. Bar graphs show means ± SD and box plots show median, boxed 25 and 75% percentiles and whiskers 10 and 90% percentiles (**a** **b**, **d**, **e**, **g**, **i**, **j**: two-tailed unpaired *t*-test; **c**, **f**: one-way ANOVA with Bonferroni post hoc comparisons; **h**: two-way ANOVA with Bonferroni post hoc comparisons; *p < 0.05, **p < 0.01, ***p < 0.001, ****p < 0.0001).

To examine whether PARP-1 is involved in the regulation of neurogenesis, we first performed neurosphere formation assay using PAPR-1 KO NSCs. As shown in Fig. 1a, PARP-1-deficient NSCs formed neurospheres with decreased number and diameter when compared with the wild-type (WT). Similar results were obtained when PARP-1 was knocked down by siRNA in the WT NSCs (Fig. 1b). The delayed neurosphere formation in the PARP-1 KO NSCs was restored when PARP-1 was overexpressed in the KO NSCs (Fig. 1c). Neurosphere formation was also retarded by PARP-1 inhibitor (Fig. 1d), suggesting PARP-1 enzymatic activity is required for the normal level of NSC proliferation or survival. Furthermore, the KO NSCs exhibited reduced BrdU incorporation, which was restored by PARP-1 reintroduction (Fig. 1e, f). PARP-1 knockdown (Fig. 1g) or inhibition of the enzymatic activity (Fig. 1h) resulted in the similar results. In addition, PARP-1 KO NSCs were more immunoreactive for the cell cycle inhibitor p27 (Fig. 1i) and p21 (Fig. 1j) when compared with the WT, suggesting that proliferation is suppressed in the PARP-1 KO NSCs. Consistently, PARP-1 inhibitor increased the expression of p27 and p21 in the WT NSCs (Fig. 1k), indicating that PARP-1 negatively regulates the expression of these cell cycle inhibitors. Taken together, these results suggest that PARP-1 is required for the normal level of NSC proliferation.

### PARP-1 is required for the survival of NSCs

**Fig. 2:**
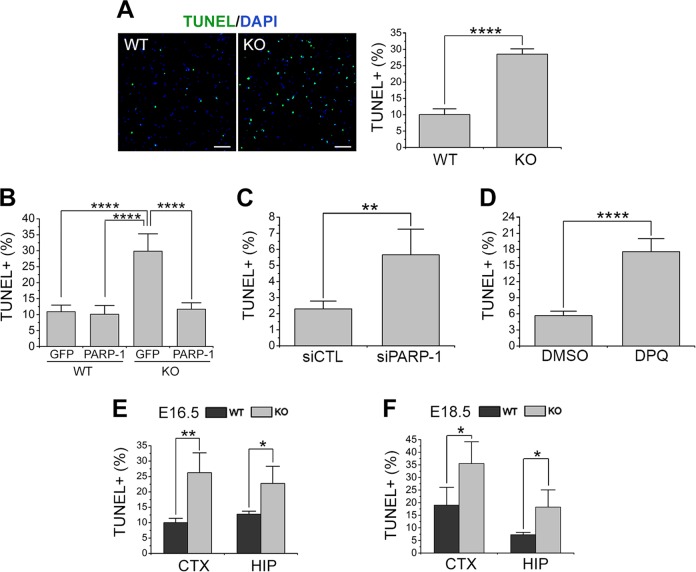
PARP-1 was required for the survival of NSCs. **a** TUNEL was performed on the NSCs prepared from PARP-1 littermate embryos at day 6 in vitro. Representative photographs are shown in the left panels (scale bar = 100 μm) and the quantification is shown in the right panel (WT, *n* = 9; KO, *n* = 10; data pooled from three independent cultures). **b** PARP-1 was reintroduced into the KO NSCs by retroviral delivery (GFP-PARP-1) and then TUNEL was performed. Infection with retrovirus carrying GFP alone served as a control (WT GFP, *n* = 4; *n* = 5 for the other groups). **c**–**d** TUNEL was performed on the control (siCTL) or PARP-1 siRNA-transfected NSCs (**c**; siCTL, *n* = 4; siPARP-1, *n* = 6) or 20 μM DPQ-treated NSCs (**d**; DMSO and DPQ, *n* = 4 each). **e**–**f** PARP-1 littermate embryonic brains were processed for TUNEL at embryonic day (ED) 16.5 in the cortex and hippocampus (HIP) (**e**; *n* = 4 each); at ED 18.5 in the cortex and hippocampus (**f**; *n* = 4 each). Means ± SD are shown (**a**, **c**, **d**, **e**, **f**: two-tailed unpaired *t*-test; **b**: one-way ANOVA with Bonferroni post hoc comparisons; *p < 0.05, **p < 0.01, ****p < 0.0001).

We then examined whether the absence of PARP-1 affects the NSC survival as well. When TUNEL assay was performed to compare the rate of spontaneous apoptosis in cultured NSCs, PARP-1 KO showed more TUNEL-positive cells (Fig. 2a), which was also reversed by reintroduction of PARP-1 (Fig. 2b). PARP-1 knockdown (Fig. 2c) or inhibition by 3,4-dihydro-5-[4-(1-piperidinyl)butoxyl]-1(2H)-isoquinolinone (DPQ) (Fig. 2d) also resulted in the increase of apoptotic cells. In a similar manner as in the cultured NSCs, PARP-1 KO brains exhibited increased number of TUNEL-positive cells at embryonic days 16.5 and 18.5 (Fig. 2e, f). These results suggest that PARP-1 is required for the survival of the NSCs as well as normal level of proliferation.

### PARP-1 negatively regulates FOXO1 activity in NSCs

**Fig. 3:**
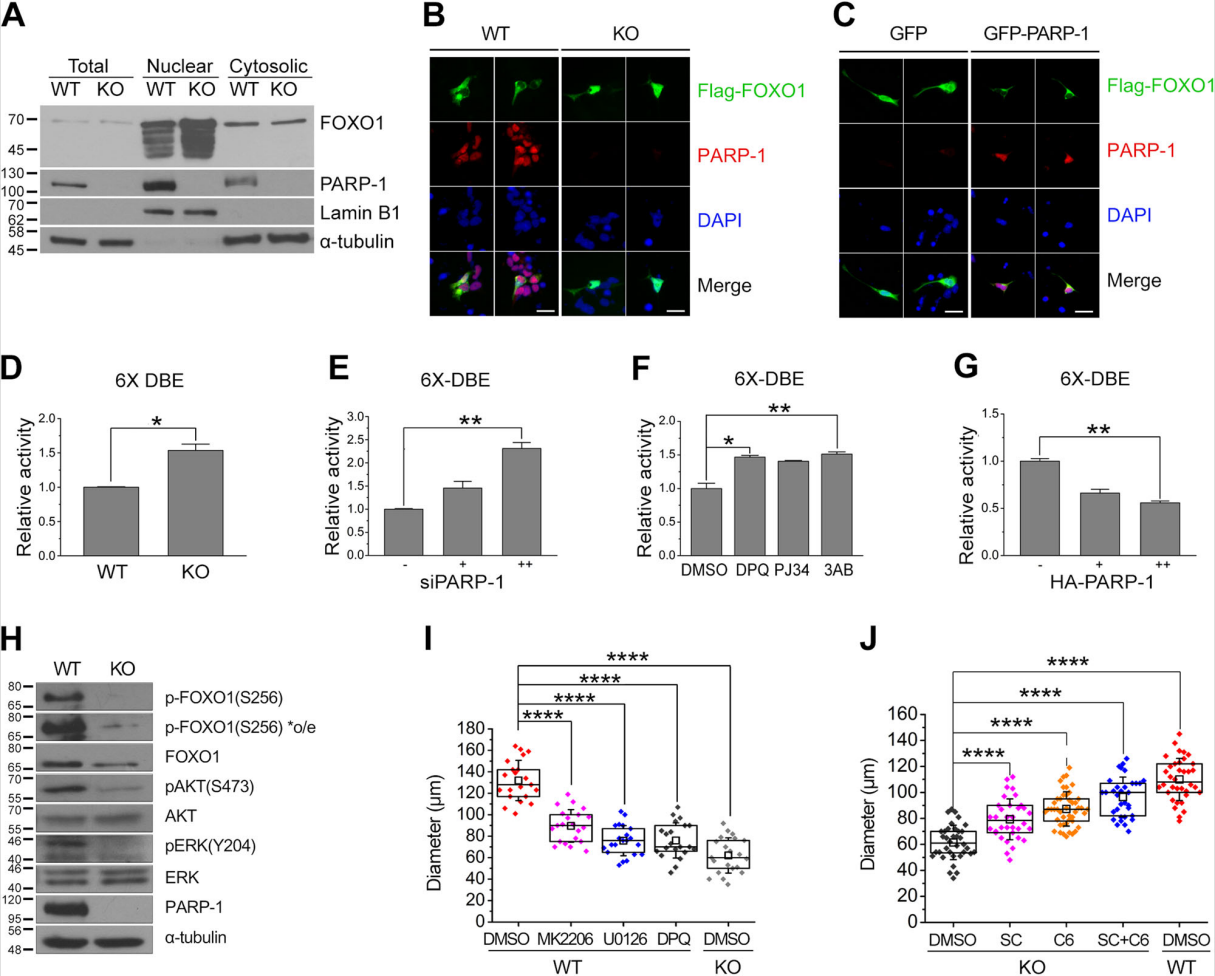
PARP-1 negatively regulated FOXO1 activity in the NSCs. **a** To assess the activation of FOXO1, PARP-1 NSCs were fractionated into nuclear and cytosolic fractions and the amount of nuclear FOXO1 was examined by immunoblots as indicated. Fractionation was confirmed by blotting with anti-lamin B1 and anti-tublin antibodies. Molecular weights on the left in kDa. **b** Cellular localization of FOXO1 was visualized by immunostaining following overexpression of Flag-FOXO1 in the PARP-1 NSCs. **c** Localization of overexpressed Flag-FOXO1 was monitored by immunostaining following retroviral reintroduction of PARP-1 in the KO NSCs (scale bar = 20 μm). **d**–**g** To examine the FOXO1 activity, FOXO1 6X DBE-luciferase was transfected with pRL-TK into PARP-1 NSCs and dual luciferase assay was performed. The FOXO1 activity was monitored in the WT and KO NSCs (**d**); in the NSCs transfected with PARP-1 siRNA (0, 50, 100 pmol) (**e**); in the NSCs incubated with PARP-1 inhibitors DPQ (50 μM), PJ34 (10 μM), or 3AB (2 mM) (**f**); in the NSCs transfected with HA-PARP-1 (**g**). **d**–**g** means ± SD are shown (**d**: Mann–Whitney test; **e**, **f**, **g**: Kruskal–Wallis test with Dunn’s post hoc comparisons; *p < 0.05, **p < 0.01; *n* = 4 independent cultures for each group). **h** Phosphorylation of Akt and ERK together with FOXO1 was examined in the WT and PARP-1 KO NSCs by immunoblotting as indicated. **i** PARP-1 NSCs were incubated to form neurospheres in the presence of MK2206 (500 nM), U0126 (5 μM), or DPQ (50 μM) and the diameters of the neurospheres were measured at day 6 (*n* = 21 for each group; data pooled from four independent cultures). **j** PARP-1 KO NSCs were incubated to form neurospheres in the presence of SC-79 (SC, 100 ng/ml), C6 ceramide (C6, 250 nM), or the two together (S + C) (KO DMSO, *n* = 36; SC, *n* = 34, C6, *n* = 46; SC + C6, *n* = 33; WT DMSO, *n* = 36; data pooled from four independent cultures). **i–j** box plots show median, boxed 25 and 75% percentiles and whiskers 10 and 90% percentiles (one-way ANOVA with Bonferroni post hoc comparisons, ****p < 0.0001).

To understand how PARP-1 regulates NSC proliferation, we then examined FOXO1 activity in PARP-1 KO NSCs. FOXO1 is regulated by phosphorylation and proteasomal degradation: when it is phosphorylated by Akt or ERK, it is localized in the cytoplasm to be degraded^27^. As shown in Fig. 3a, PARP-1 KO NSCs contained more FOXO1 in the nuclear fraction than the WT. When FOXO1 was overexpressed in the NSCs, the WT cells showed mostly cytoplasmic localization of the exogenous FOXO1 while KO cells retained FOXO1 in the nucleus as well (Fig. 3b). When we overexpressed PARP-1 in the KO NSCs, FOXO1 was now found in the cytoplasm in the PARP-1 expressing cells but not in the PARP-1-deficient cells (Fig. 3c). These results imply that PARP-1 negatively regulates FOXO1 by inducing its cytoplasmic localization. In addition, FOXO1 transcriptional activity was increased in the PARP-1 KO NSCs (Fig. 3d). Consistently, knockdown or inhibition of PARP-1 in the WT NSCs enhanced the FOXO1 activity (Fig. 3e, f). Overexpression of PARP-1 in the KO NSCs, however, suppressed the FOXO1 transcriptional activity (Fig. 3g).

Then we examined the phosphorylation status of FOXO1 in the PARP-1 KO cells. Figure 3h shows that FOXO1 phosphorylation was much reduced in the PARP-1-deficient NSCs consistently with the FOXO1 localization and activity data. Moreover, phosphorylation of upstream kinases including Akt and ERK was reduced in the PARP-1 KO cells (Fig. 3h). When NSCs were incubated with the inhibitors of Akt (MK2206) or ERK (U0126), neurosphere diameters were reduced to the level when PARP-1 was absent or inhibited by DPQ (Fig. 3i). Importantly, activators of Akt (SC-79) or ERK (C6 ceramide) reversed the decrease in the diameter of the PARP-1 KO neurospheres (Fig. 3j). Taken together, these results suggest that PARP-1 is required for the suppression of FOXO1 activity and activation of Akt and ERK.

### PARP-1 negatively regulates the expression of embryonic stem cell phosphatase

To understand the mechanism how PARP-1 regulates NSC proliferation, we examined the changes in the gene expression profile between the WT and KO NSCs by microarray analysis. A hierarchical clustering revealed that 17,549 genes were changed more than two-folds (Fig. S1A). We focused our attention on phosphatases that may suppress Akt signaling. Interestingly, many receptor type-protein tyrosine phosphatases were upregulated in the PARP-1 KO NSCs (Fig. S1B). Among those, the expression of embryonic stem cell phosphatase (ESP, also known as protein tyrosine phosphatase receptor type V) was increased more than 40-fold in the KO NSCs (Fig. S1B).

**Fig. 4:**
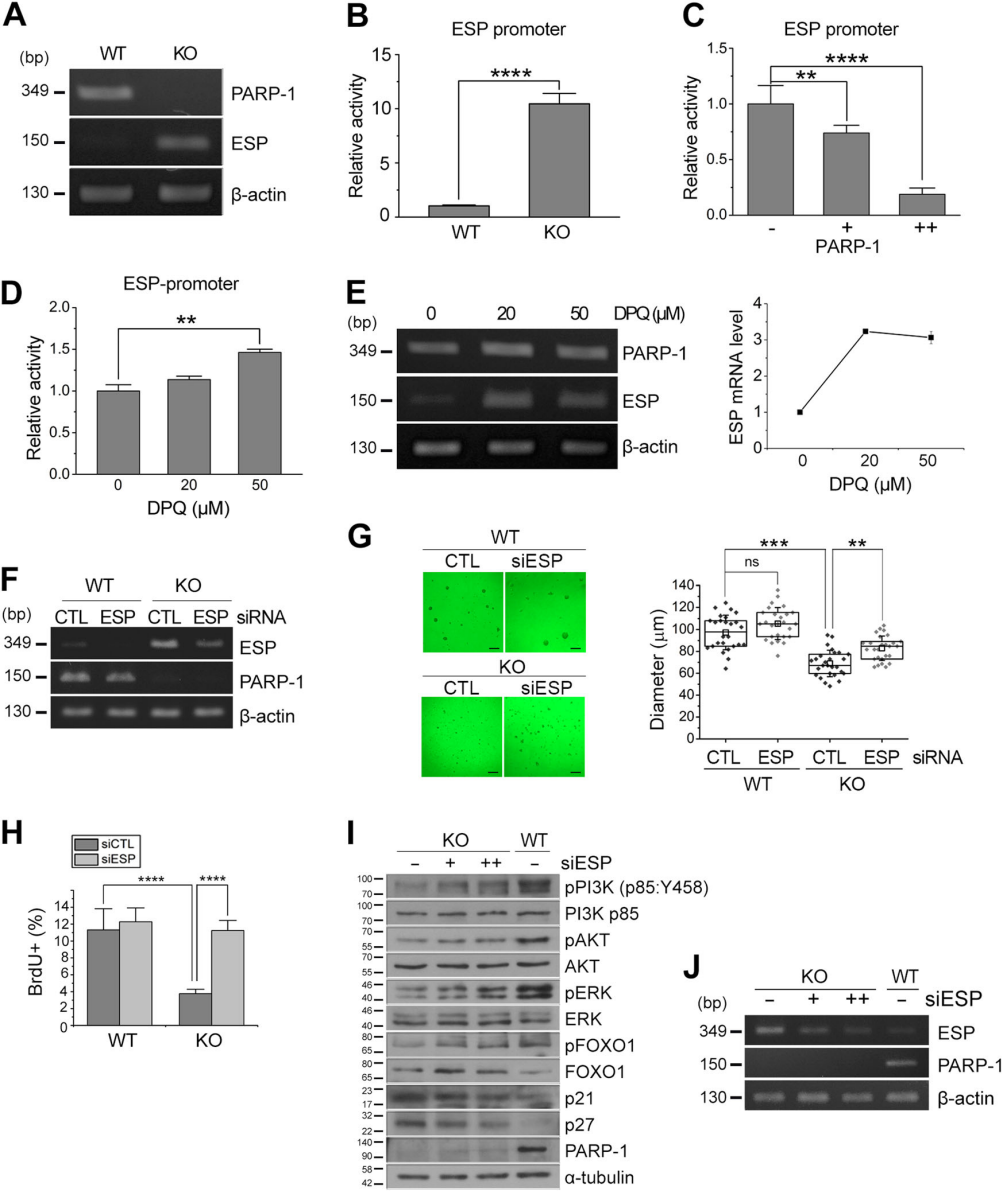
PARP-1 negatively regulated the expression of ESP. **a** mRNA level of ESP was examined in the PARP-1 WT and KO NSCs by RT-PCR. β-Actin served as an internal control. **b**–**d** ESP promoter-luciferase activity was determined in the PARP-1 NSCs (B; *n* = 5); in the NSCs following overexpression of PARP-1 (**c**: *n* = 5); in the NSCs incubated with DPQ (**d:** *n* = 4). **e** mRNA level of ESP after DPQ treatment was determined in the NSCs by RT-PCR. **f–h** PARP-1 NSCs were incubated to form neurospheres following transfection of siRNA for ESP. Knockdown of ESP was confirmed by RT-PCR (**f**). **g** Representative images of ESP-knocked down NSCs are shown (scale bar = 200 μm) and the quantification of the neurosphere diameter is shown in the right panel (WT, *n* = 26 each; KO, *n* = 27 each; data pooled from four independent cultures). **h** PARP-1 NSCs were transfected with ESP siRNA and then the rate of proliferation was monitored by BrdU labeling (WT CTL, *n* = 7; WT siESP, *n* = 6; KO CTL, *n* = 15; KO siESP, *n* = 7; data pooled from two independent cultures). **i**–**j** Activation of PI3K, Akt, and ERK was examined by immunoblotting following knockdown of ESP in the PARP-1 KO NSCs. The membranes were reprobed to examine the changes in the level of p21, p27 and phospho-FOXO1. A blot with anti-tubulin served as a loading control. Molecular weights on the left in kDa (**i**). ESP knockdown was confirmed by RT-PCR (**j**). Bar graphs show means ± SD and box plots show median, boxed 25 and 75% percentiles and whiskers 10 and 90% percentiles (**b**: two-tailed unpaired *t*-test; **c**, **g**: one-way ANOVA with Bonferroni post hoc comparisons; **d**: Kruskal–Wallis test with Dunn’s post hoc comparisons; **h**: two-way ANOVA with Bonferroni post hoc comparisons; **p < 0.01, ****p < 0.0001).

A previous study reported that ESP is a direct target of p53 and mediates p53-induced cell cycle arrest, implying that ESP is an inhibitor of cell proliferation^28,29^. However, it has not been studied whether ESP is involved in the regulation of proliferation or differentiation of the stem cells. To examine the possible involvement of ESP in the PARP-1-regulated NSC proliferation, we first confirmed the upregulation of ESP in the PARP-1 KO NSCs. An RT-PCR analysis revealed that mRNA level of ESP was markedly increased in the PARP-1-deficient NSCs (Fig. 4a). Consistently, the activity of ESP promoter was significantly increased in the KO cells (Fig. 4b). Reintroduction of PARP-1 in the KO NSCs suppressed the ESP promoter activity very efficiently (Fig. 4c). Conversely, addition of PARP-1 inhibitor DPQ enhanced the ESP promoter activity (Fig. 4d) and increased the mRNA level of ESP (Fig. 4e) in the WT NSCs. These results indicate that PARP-1 negatively regulates the transcription of ESP.

To address whether the decreased proliferation of PARP-1 KO NSCs is due to the ESP upregulation, we then knocked down the expression of ESP in the PARP-1 KO NSCs and monitored the neurosphere formation. As shown in Fig. 4f, g, knockdown of ESP increased the diameter of PARP-1 KO neurospheres. Consistently, the rate of proliferation as assessed by BrdU incorporation was also restored following ESP knockdown in the PARP-1 KO NSCs (Fig. 4h).

We then examined whether the increase in ESP expression is responsible for the decrease in Akt or ERK activation in the PARP-1-KO NSCs. As shown in Fig. 4i, j, the ESP knockdown resulted in the increase of Akt and ERK phosphorylation suggesting that the forced suppression of ESP expression in the PARP-1-deficient cells restored the activity of Akt and ERK. Because ESP is a receptor type tyrosine phosphatase^30^ and Akt or ERK is activated by serine/threonine phosphorylation, we hypothesized that ESP may inhibit upstream kinase activated by tyrosine phosphorylation. Thus we examined whether PI3K is involved. As expected, activation of PI3K was much suppressed in the PARP-1 KO NSCs and knockdown of ESP enhanced the activation of PI3K (Fig. 4i, j). Consistently, expression of p21 and p27 was also reduced by ESP knockdown (Fig. 4i, j). Taken together, these results suggest that PARP-1 maintains the normal level of PI3K/Akt-ERK activity by repressing the expression of ESP, a suppressor of PI3K signaling.

### PARP-1 is required for the normal level of neuronal differentiation of the NSCs

**Fig. 5:**
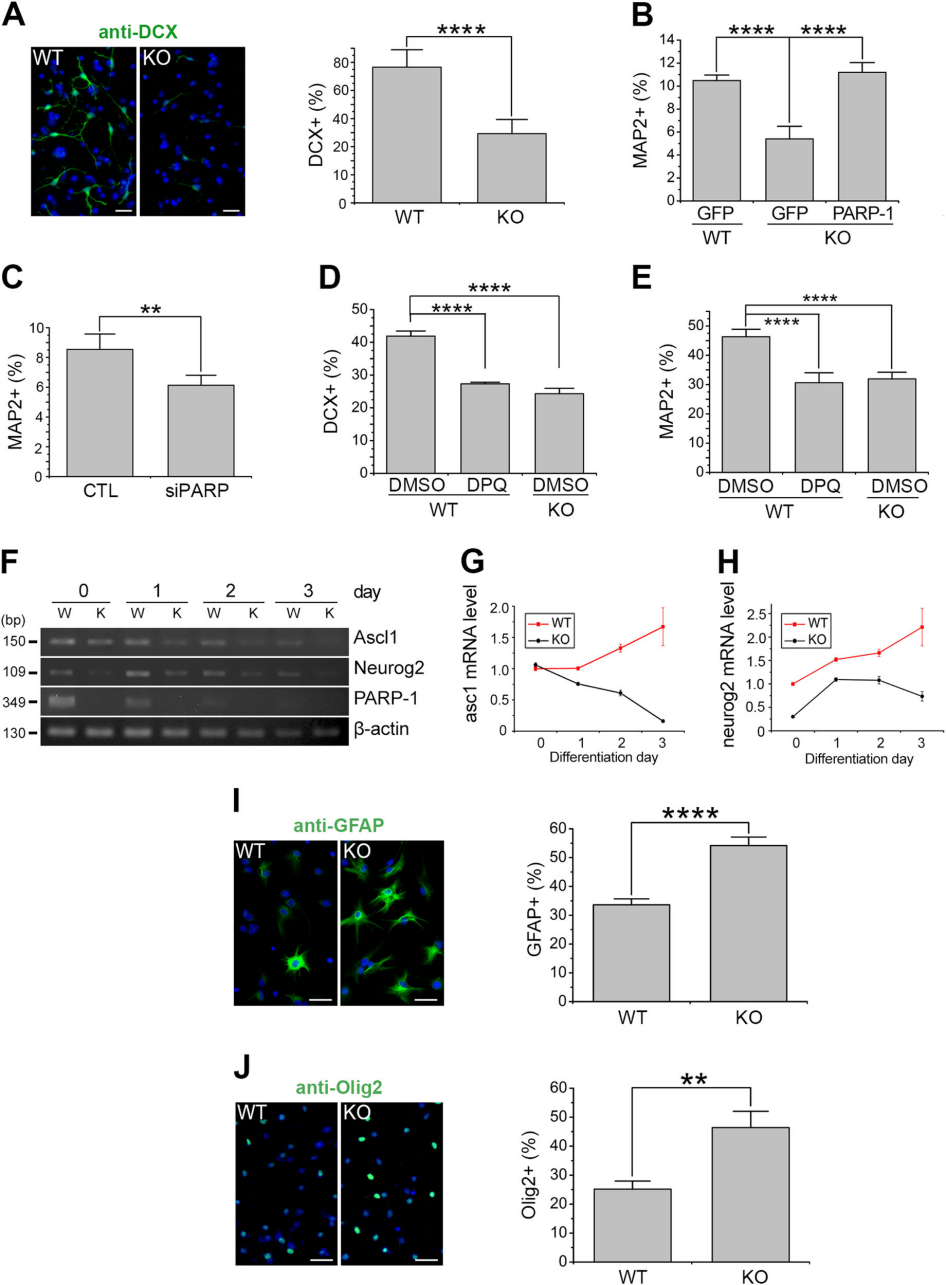
PARP-1 was required for the normal level of neuronal differentiation of the NSCs. **a** PARP-1 NSCs were grown in the differentiation medium and then immunostained with anti-DCX at day 3. Representative images are shown in the left (scale bar = 20 μm). The DCX-positive cells were counted and quantified (right panel; *n* = 8 WT, *n* = 12 KO; data pooled from four independent cultures). **b** PARP-1 KO NSCs were infected with retrovirus carrying PARP-1, differentiated and then immunostained for MAP2 and quantified at day 5 (*n* = 5, pooled from three independent cultures). **c** NSCs were transfected with siRNA for PARP-1, differentiated and then immunostained for MAP2 (*n* = 5; pooled from three independent cultures). **d**–**e** NSCs were differentiated in the presence of DPQ (50 μM) and then processed for immunocytochemistry using anti-DCX at day 3 (**d:** WT DMSO, *n* = 6; WT DPQ, *n* = 3; KO DMSO, *n* = 5; pooled from two independent cultures) and anti-MAP2 antibodies at day 6 (**e:** WT DMSO, *n* = 7; WT DPQ, *n* = 6; KO DMSO, *n* = 9; pooled from two independent cultures). **f**–**h** PARP-1 NSCs were cultured in the differentiation medium and processed for RT-PCR as indicated (**f**). Relative mRNA expression levels of ASCL1 (**g**) and Ngn2 (**h**) were quantified by densitometry. **i**–**j** PARP-1 NSCs were cultured in the differentiation medium and then immunostained for GFAP at day 5 (**i:** WT, *n* = 6; KO, *n* = 6; pooled from three independent cultures) and for Olig2 at day 2 (**j**: WT, *n* = 4; KO, *n* = 3; pooled from two independent cultures). Representative photographs are shown on the left (scale bar = 20 μm) and the quantification, on the right. Means ± SD are shown (**a**, **c**, **i**, **j**: two-tailed unpaired *t*-test; **b**, **d**, **e**: one-way ANOVA with Bonferroni post hoc comparisons; **p < 0.01, ****p < 0.0001).

A previous study reported that PARP-1 deletion promotes postnatal subventricular zone (SVZ) NSC differentiation into oligodendrocytes^31^. Thus we further examined whether PARP-1 can regulate the differentiation of NSCs. The immunostaining analysis revealed that the number of doublecortin (DCX)- or microtubule-associated protein 2 (MAP2)-positive cells was decreased by ~50% in the PARP-1 KO cells (Fig. 5a, b). The ratio of MAP2-positive cells was restored after overexpression of PARP-1 in the KO NSCs (Fig. 5b). The number of cells expressing neuronal markers was also decreased after PARP-1 knockdown (Fig. 5c) or pharmacological inhibition (Fig. 5d, e). To further confirm whether PARP-1 functions in the neuronal differentiation, we examined the changes in the neurogenic transcription factors in the KO NSCs by RT-PCR. Consistently with the decreased neurogenesis, the mRNA levels of neurogenic factors such as achaete-scute homolog 1 (ASCL1) and neurogenin 2 were decreased in the PARP-1 KO NSCs (Fig. 5f-h).

We then investigated whether PARP-1 affects the glial differentiation. As shown in Fig. 5i, j, the PARP-1 KO cells exhibited increased number of astrocyte marker glial fibrillary acidic protein (GFAP)- or oligodendrocyte marker Olig2-positive cells under differentiation condition. Consistently, when we examined the expression of nestin, Tbr2 (an intermediate neural progenitor marker) and Olig2 in embryonic day 16.5 brains, we observed a similar tendency as in cultured NSCs, i.e., decreased neurogenesis and increased oligodendrogenesis in PARP-1 KO (Fig. S2). These results suggest that PARP-1 is required for the normal level of neurogenesis.

### PARP-1 KO mice exhibited reduced brain weight and decreased proliferation of adult NSCs

**Fig. 6:**
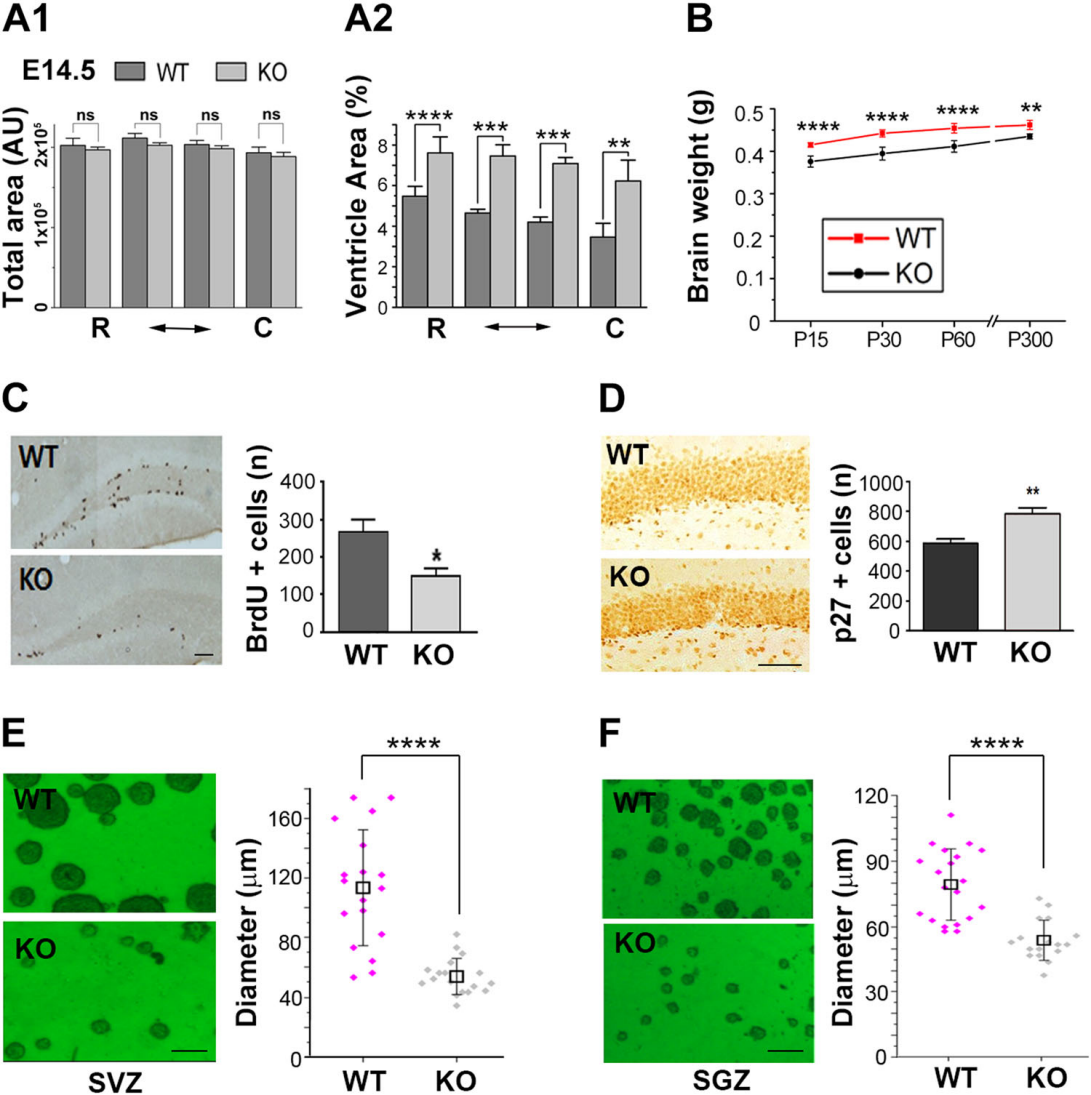
PARP-1 KO mice exhibited reduced brain weight and decreased proliferation of adult NSCs. **a** Total brain area (A1) and ventricle area were measured from Nissl-stained brain sections of PARP-1 littermates at ED 14.5 and the percent cross-sectional ventricle area over total brain area (A2) was quantified (*n* = 5 for each group). **b** Brain weights of littermates were measured at postnatal day 15 (WT, *n* = 12; KO, *n* = 12), day 30 (WT, *n* = 9; KO, *n* = 13), 60 (WT, *n* = 7; KO, *n* = 7) and day 300 (WT, *n* = 5; KO, *n* = 4). **c** Immunolocalization of proliferating adult progenitor cells in the hippocampal dentate gyrus from WT (*n* = 5) and PARP-1 KO (*n* = 4) mice (scale bar = 50 μm) and quantitative analysis of BrdU-positive cells. **d** Immunohistochemical localization of p27-positive cells in the dentate gyrus from WT and PARP-1 KO mice and quantitative analysis of p27-positive cells (*n* = 4 for each group). **e**, **f** Neurosphere cultures derived from the adult SVZ (**e**) and SGZ (**f**) of WT and PARP-1 KO mice (scale bar = 100 μm) and the sizes of neurospheres were compared (**e**; WT, *n* = 38; KO, *n* = 33, **f**; WT, *n* = 40, KO, *n* = 35; pooled from four independent cultures). Means ± SD are shown (*p < 0.05, **p < 0.01, ****p < 0.0001, two-tailed unpaired *t*-test).

To further investigate whether PARP-1 is required for normal brain development, we examined the cross-sectional area of ventricle and the brain weights. As shown in Fig. 6a, ventricle area over total coronal cross-section area was significantly increased in the PARP-1 KO brains at embryonic day 14.5. Furthermore, the KO mice exhibited reduced brain weights throughout the early postnatal period and adulthood (Fig. 6b). When we examined anatomical structures of the adult hippocampus and the cortex by NeuN and GFAP immunohistochemistry, we could not find any noticeable difference between WT and KO (Fig. S3). However, there was a tendency of decrease of NeuN-positive neurons in the cortex of the PARP-1 KO mice although it did not reach statistical significance (Fig. S3).

Then we examined whether PARP-1 regulates proliferation of NSC during adult stage as well. The number of BrdU-positive cells in the adult hippocampal dentate gyrus was less abundant in PARP-1 KO mice (Fig. 6c). Furthermore, PARP-1 KO mice exhibited a significantly increased number of p27-positive cells in the hippocampal subgranular zone (SGZ) (Fig. 6d). Consistently, the decreased number of Ki67-positive cells was observed in the hippocampus of the PARP-1 KO (Fig. S4A). The number of positive cells for a NSC marker, SOX2, was also decreased in the PARP-1 KO (Fig. S4B). The decreased proliferation of NSCs in the absence of PARP-1 may lead to decreased adult neurogenesis: NeuroD1- or DCX-positive cells were also decreased in the hippocampus of the PARP-1 KO (Fig. S5). We obtained a consistent result in the neurosphere formation assay using adult hippocampal NSCs. As in the embryonic stage (Fig. 1a), adult hippocampal NSCs prepared from SVZ or SGZ of the PARP-1 KOs formed much smaller neurospheres (Fig. 6e, f), suggesting PARP-1 is necessary for the normal level of proliferation in adult NSCs.

### Behavioral deficits in PARP-1 KO mice

**Fig. 7:**
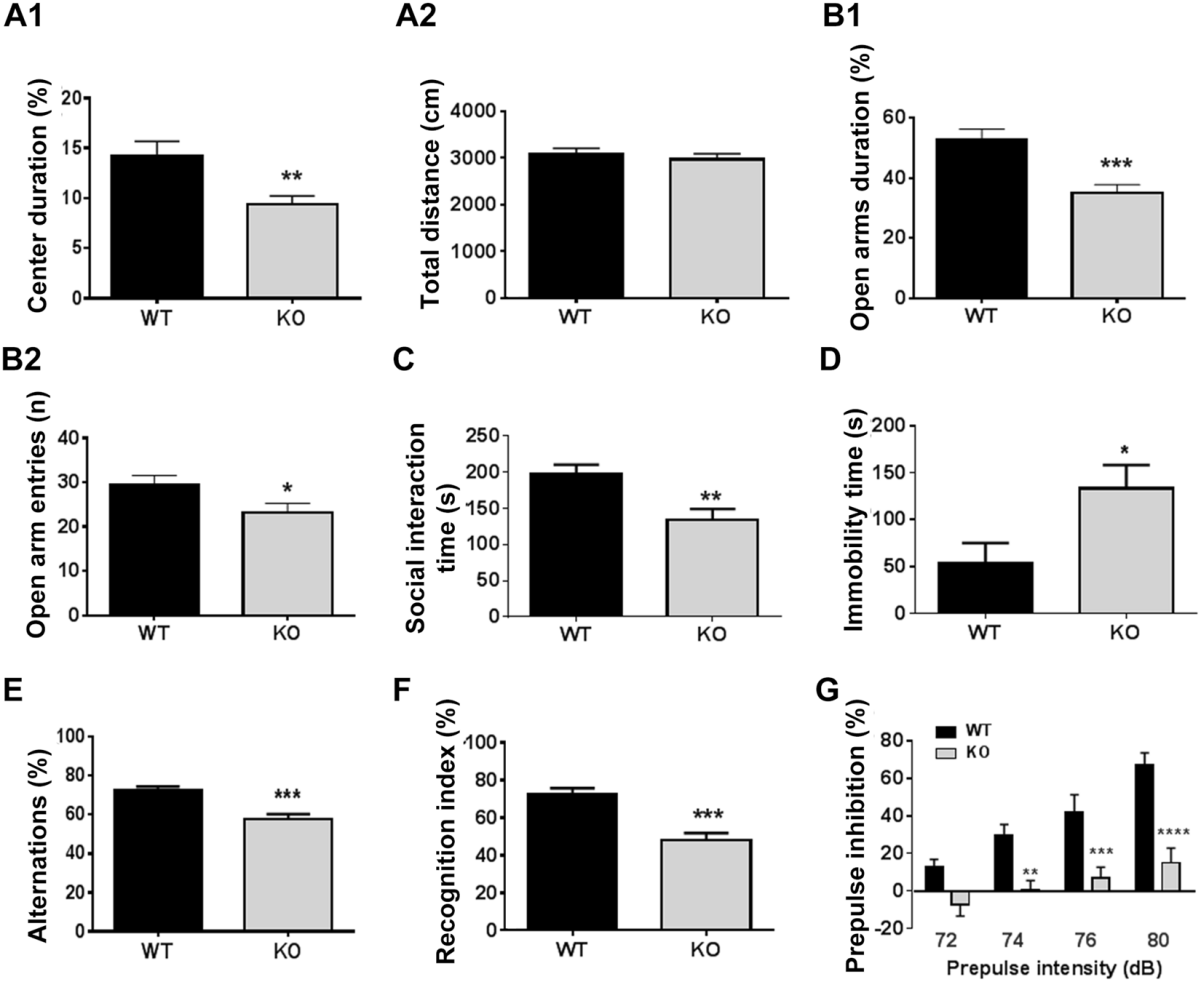
PARP-1 KO mice exhibited schizophrenia-like behavioral deficits. **a** The open field test showed that the time spent in the center was significantly longer in PARP-1 KO mice compared to WT mice (**a**1; WT: 14.91 ± 1.48%, *n* = 46; KO: 9.36 ± 0.86%, *n* = 46 animals). The total moving distance was not different (**a**2; WT: 3084 ± 123.3 cm, *n* = 46; KO: 2969 ± 119.5 cm, *n* = 46 animals). **b** The duration in open arms of PARP-1 KO mice was significantly lower than that of WT (B1; WT: 52.77 ± 3.53%, *n* = 25; KO: 34.97 ± 2.78%, *n* = 26 animals). PARP-1 KO mice visited open arms less frequently compared to WT mice (WT: 29.56 ± 2.02%, *n* = 25; KO: 23.12 ± 2.14%, *n* = 26 animals). **c** In the social interaction test PARP-1 KO mice spent less time with a stranger mouse compared to WT mice (WT: 197.0 ± 13.59 s, *n* = 14 animals; KO: 133.9 ± 15.13 s, *n* = 12 animals). **d** PARP-1 KO mice showed a significantly increased immobility in the forced swim test (WT: 54.0 ± 20.87 s, *n* = 5 animals; KO: 133.3 ± 24.81 s, *n* = 7 animals). **e** PARP-1 KO mice showed a significant decrease in percent alternation in the Y-maze test (WT: 72.21 ± 2.092%, *n* = 20 animals; KO: 57.46 ± 2.771%, *n* = 15 animals). **f** PARP-1 KO mice showed little preference toward the novel object (WT: 72.50 ± 3.26%, *n* = 11 animals; KO: 48.04 ± 3.84%, *n* = 11 animals). **a**–**f:** means ± SEM are shown (*p < 0.05, **p < 0.01, ***p < 0.001, two-tailed unpaired *t*-test). **g** PARP-1 KO mice exhibited a significant PPI deficit at multiple intensities of prepulse stimuli (*n* = 10 animals for each group). Two-way repeated measures ANOVA detected a significant effect of genotype (*F*_1,16_ = 22.29, *P* = 0.0002), prepulse intensity (*F*_3,48_ = 26.38, *P* < 0.0001) and interaction between these factors (*F*_3,48_ = 4.74, *P* = 0.0057). Bonferroni’s post hoc comparisons revealed that PARP-1 KO mice differed significantly from wild-type mice at the 74 dB (**p < 0.01), 76 dB (***p < 0.001), and 78 dB (****p < 0.0001) prepulse intensities. Means ± SEM are shown.

In order to assess whether PARP-1 KO mice present behavioral alterations, we subjected them to a battery of cognitive and emotional tests. We found that PARP-1 KO mice display emotional and cognitive deficits associated with schizophrenia-like behaviors. The open field test was used to examine the locomotor/exploratory and anxiety-related behaviors. The time spent in the center area was significantly longer in PARP-1 KO mice compared to WT mice (Fig. 7a1). However, no significant change in the total moving distance was detected (Fig. 7a2), suggesting the higher anxiety of PARP-1 KO mice without any changes in locomotor activity. Consistently, the elevated plus maze test also showed that PARP-1 KO mice exhibited high anxiety, i.e., decrease in open arm duration (Fig. 7b1) and open arm entries (Fig. 7b2). Furthermore, PARP-1 KO mice showed deficient social interaction (Fig. 7c) and depression-like behavior as judged by the increased immobility in forced swim test (Fig. 7d).

For testing the cognitive capability of PARP-1 KO mice, Y-maze spontaneous alternation and novel object recognition tests were performed. PARP-1 KO mice exhibited impaired long-term recognition memory in addition to impaired spatial working memory. The Y-maze test revealed impaired spatial working memory in PARP-1 KO mice (Fig. 7e). Moreover, PARP-1 KO mice showed little preference for the novel object (Fig. 7f).

A sensorymotor gating deficit, as measured by the prepulse inhibition of the startle response is used as a clinical measure of schizophrenia or anxiety disorder. As shown in Fig. 7g, PARP-1 KO mice exhibited a significant PPI deficit at multiple intensities of prepulse stimuli. Intriguingly, we found that PPI deficit in PARP-1 KO animals was restored by haloperidol, a blocker of dopamine D_2_ receptor (Fig. S6). Therefore, the altered neurodevelopment caused by PARP-1 deficiency may result in the dopaminergic hyperactivity, which produces, albeit partly, schizophrenia-like behaviors in PARP-1 KO mice. General health as assessed by moving velocity and skeletal muscle strength was not impaired in the PARP-1 KO mice (Fig. S7).

## Discussion

In the present study, we present evidence that PARP-1 regulates proliferation and differentiation of NSCs and its absence results in defective neurogenesis and behavioral deficits reminiscent of schizophrenia in mice. It has been suggested that neurodevelopmental defects can be a cause of psychiatric disorders including schizophrenia^32,33^. Earlier studies suggested that ventricle enlargement in the schizophrenic patients is the evidence that links defective neurodevelopment and schizophrenia^34,35^. In addition, decrease in cortical gray matter in the patients' brain has been reported thereafter^36^. Recent studies using mice transgenic or knockout for the genes associated with schizophrenia have further supported the neurodevelopmental hypothesis of schizophrenia. Most extensively studied are the DISC1 transgenic mice^37^. DISC1 was identified as one of the schizophrenia-associated genes and found to be disrupted by chromosomal translocation, truncated or point-mutated in patients with schizophrenia^38^. Depending on the interacting partners^39^, DISC1 activity regulates distinct signaling pathways. Through the interaction with KIAA1212, DISC1 inhibits Akt activity, which in turn regulates differentiation of postmitotic newborn neurons^12^. In NSCs, instead, DISC1 regulates proliferation through inhibition of GSK3β, a downstream target of Akt^11^. Accordingly, DISC1 and Dixdc1 interact to regulate NSC proliferation by modulating Akt-GSK3β signaling^40^. Therefore, DISC1 functions in the regulation of NSC proliferation, neuronal differentiation, and neuronal migration during brain development^11,41–44^.

The regulation of NSC proliferation by PARP-1 turned out to be mediated by Akt and ERK signaling converging on FOXO1. Apart from the involvement in the schizophrenia pathogenesis downstream of DISC1, Akt has been independently suggested as a schizophrenia susceptibility gene in the genetic linkage and association studies^19^. In accordance with this, the protein levels and activity of Akt decreased in the schizophrenic patients^19,20^. In our observation, Akt activity was markedly suppressed in the absence of PARP-1 and pharmacological activator of Akt restored the cell proliferation defect in the PARP-1 KO NSCs. Our results further support the involvement of Akt signaling in neurodevelopmental defects associated with schizophrenia.

As a mechanism behind the lowered Akt activity in the absence of PARP-1, we found that the expression of ESP, a receptor type protein tyrosine phosphatase, was strikingly elevated in the PARP-1 KO NSCs. ESP has been considered as a tumor suppressor candidate since it mediates the p53-induced cell cycle exit^29,30^. We found that PARP-1 negatively regulates the transcription of ESP via PARP enzymatic activity. As to the mechanism of PARP-1-mediated ESP repression, one possibility is that PARP-1 suppresses the transcription of ESP by inhibiting p53 activity. A recent study suggested that a PARP-1 inhibitor activates p53 by ATM/ATR-mediated phosphorylation in NSCs^45^. Furthermore, ESP is known to be a direct target gene of p53^29^. Therefore, it is highly possible that PARP-1 represses the transcription of ESP via p53 regulation. However, it cannot be ruled out that PARP-1 inhibits the activity of essential transcription factors of the ESP by direct poly ADP-ribose (PAR) modification.

The role of PARP-1 was extended to the regulation of NSC differentiation as well: the rate of neuronal differentiation was decreased while that of astrocytic and oligodendrocytic differentiation was increased in the absence of PARP-1 in the cultured NSCs. Plane and colleagues previously reported that the percentage of oligodendrocyte progenitor cells (OPCs) increased while that of neuroblasts decreased in the SVZ of postnatal day 11 PARP-1 KO mice^31^. In spite of the increased number of OPCs, the layer of mature and myelinating oligodendrocytes in corpus callosum of PARP-1 KOs was thinner than that of WT^31^. They suggested that the increase of OPCs may be a compensatory mechanism for the myelination deficiency. We also observed the increase in the percentage of glial differentiation in the embryonic NSCs. However, oligodendrocytic differentiation was increased in the absence of PARP-1 even in the embryonic NSCs in culture irrespective of myelination in our observation. Our data indicate that PARP-1 actively regulates neuronal differentiation of NSCs: Basic helix-loop-helix transcription factors required for the neuronal differentiation were downregulated in the PARP-1 KO NSCs. Specifically, expression of ASCL1 and neurogenin 2 was significantly suppressed in the PARP-1 KO NSCs (Fig. 5). However, expression of oligodendrogenic transcription factors was not much altered in the PARP-1 KO NSCs: fold-changes of Olig 1 and Olig2 expression in the KO were 1.25 and 1.114 in our microarray analysis, respectively. Therefore it can be postulated that the increased gliogenesis may be due to decreased neurogenesis. Previously, it has been reported that PARP-1 is complexed with TLE to repress the transcription of ASCL1 but upon activation by calcium signaling, it derepresses ASCL1 transcription by PARylating and driving off TLE^26^. Our results indicate that PARP-1 regulates the expression of not only a single neurogenic factor following calcium signaling but also multiple neurogenic factors even before the differentiation stage.

PARP-1 KO mice showed neurodevelopmental and behavioral deficits very similar with the phenotypes reported in the patients and the mouse models of schizophrenia. We found that PARP-1 KO mice exhibited enlarged ventricle during development and reduced brain weight in embryos and adults. Our results suggest that the reduced brain mass may result from the decreased neurogenesis of the NSCs. Interestingly, no remarkable structural difference was found in the hippocampus and the somatosensory cortex of the adult PARP-1 KO mice although there is a tendency of decrease in the cortical NeuN-positive neurons in the PARP-1 KO. Therefore, more detailed characterization of the regional structural difference of the brain between WT and PARP-1 KO mice is needed to fully understand the neurodevelopmental defect associated with schizophrenia-like behaviors in the PARP-1 KO mice.

In conclusion, we found that PARP-1 regulates the proliferation and differentiation of NSCs by orchestrating the expression of key regulators of neurogenesis. The defective neurogenesis in the absence of PARP-1 during development and throughout the adulthood may be a cause of multiple behavioral abnormalities found in schizophrenia including anxiety, depression and deficits in memory, social interaction, and PPI.

## Materials and methods

### Animals

Animal maintenance and treatment were performed in accordance with the Animal Care and Use Guidelines issued by Kyung Hee University and Sejong University. All experiments with mice were carried out according to the protocols approved by the Institutional Animal Care and Use Committees of Kyung Hee University (approved protocol No. KHU(SE)-13-031, KHU(SE)-15-026) and Sejong University (approved protocol No. SJ-20120901-E2). PARP-1 KO mice (129S-PARP1^tm1Zqw^, Jackson Laboratory) were backcrossed onto C57BL/6J strain more than eight times. Matched littermates of PARP-1 KO and WT mice were analyzed. Mice were group-housed on a 12 h:12 h light:dark cycle and had free access to food and water. The animals were held in a chamber at 20–24 °C with 30–60% humidity.

### Cell culture and sphere formation assay

Embryonic NSCs were cultured from E13.5 C57BL/6J or PARP-1 mice cerebrocortex. Briefly, the neocortices of mouse embryos on embryonic day 13.5 were dissected, cut into small pieces and mechanically triturated in cold PBS. The dissociated cells were collected by centrifugation for 5 min at 1000 × *g* and resuspended in Dulbecco's Modified Eagle's Medium (DMEM): F12 (1:1) medium (Gibco) supplemented with 2% B-27 (Gibco), 20 ng/mL epidermal growth factor (EGF) and 20 ng/mL basic fibroblast growth factor (FGF) (R&D systems). Cells were plated on untreated petri dishes in the culture medium and incubated with 5% CO_2_ at 37 °C. The culture medium was changed every 3−4 days. After 5−7 days, the cells were mechanically dissociated and replated in a new culture flask at a density of 1 × 10^5^ cells/mL with fresh culture medium. Adult NSCs were isolated from 8–12 week-old mouse SVZ or SGZ. For the sphere formation assay, primary cultured cells or serially passaged cells were resuspended with DMEM: F12 (1:1) and the number of viable cells was counted by trypan blue exclusion assay in a hemocytometer (Marienfeld). Cells were plated onto uncoated 96 well-plate at a density of 3 × 10^3^ cells/well in the growth medium. The cells were incubated with 5% CO_2_ at 37 °C. Three to five days after seeding, the number of spheroids and their diameters were measured under a microscope. For embryonic NSC cultures, NSCs from the brain of an individual littermate embryo were cultured separately and then genotyped. After genotyping, cells from 2–3 embryos of the same genotype were pooled for further experiments. For adult NSC cultures, cells from at least three brains of the same genotype were pooled. For the experiments using cultured NSCs, cultures were randomly assigned to the treatments and data points were pooled from 2–4 independent experiments.

### Retroviral infection and siRNA transfection

For retroviral infection, 10^4^ cells were incubated with viral suspension at a volume of 3% of the plating media. One microliter of the viral suspension contained 1 × 10^4^ viral particles. Three days after the infection, infection was confirmed by GFP expression. Human PARP-1 siRNA (5′-CUCUCAAAUCGCUUUUACA-3′), mouse PARP-1 siRNA (5′-GGACCGAAUAUUCCCUCCA-3′) and negative control siRNA were purchased from Bioneer. siRNA (50–150 pmol) was delivered using the Lipofectamine 2000 or LTX with Plus (Life Technologies) according to the manufacturer's protocol.

### Antibodies

The following antibodies were used: mouse monoclonal anti-PARP-1, mouse monoclonal anti-BrdU (#347580), mouse monoclonal anti-p21 (#556431), mouse monoclonal anti-p27 (#610241), rabbit polyclonal anti-PAR (#551813,; BD Bioscience), mouse monoclonal anti-α-tubulin (#T5168, Sigma-Aldrich), mouse monoclonal antibodies for Myc (#G019), HA (#G036), Flag (#G191), and GFP (#G096, Abm), rabbit polyclonal anti-MAP2 (#MAB3418), anti-Olig2 (#ab9610), anti-SOX2 (#ab5603, Millipore), anti-GFAP (#ab7260), anti-Ki67 (#ab15580), anti-Tbr2 (#ab183991) and mouse monoclonal anti-Nestin (#ab22035, abcam), goat polyclonal anti-DCX (#sc-8066), anti-NeuroD (#sc-1084), rabbit polyclonal anti-ERK (#sc-93), mouse monoclonal anti-pERK (#sc-7383), anti-LaminB1 (#sc-56145, Santa Cruz), rabbit polyclonal anti-Akt (#4060), rabbit polyclonal anti-pAkt (#4061), rabbit polyclonal anti-PI3K (#4228), anti-pPI3K (#4257), rabbit polyclonal anti-FOXO1 (#2880), rabbit polyclonal anti-pFOXO1 antibody (#9461, Cell Signaling).

### Terminal deoxynucleotidyl transferase dUTP nick end labeling assay

To detect apoptotic cells, terminal deoxynucleotidyl transferase dUTP nick end labeling (TUNEL) was performed with ApopTag Fluorescein In Situ Apoptosis Detection Kit (Millipore) according to the manufacturer's protocol.

### Luciferase assay

To measure FOXO1 activity or ESP promoter activity, NSCs cells were transfected with expression vectors encoding PARP-1 or siPARP-1, along with the 6XDBE- or ESP-luciferase reporter vector (kindly provided by Dr. Gerard Karsenty, Columbia University, USA) and control pRL-TK plasmids. When required, the cells were incubated with 50 μM of DPQ for 16 h prior to analysis. Dual luciferase activity assay was carried out using dual luciferase reporter assay kit (Promega) according to the manufacturer’s protocol.

### Subcellular fractionation

Cells (5 ×10^6^) were incubated in the cytosolic fractionation buffer (10 mM HEPES, 50 mM NaCl, 0.1 mM EDTA, 0.5 M Sucrose, 0.3% Triton X-100, 1 mM DTT, 1 mM PMSF, 10 μg/ml Leupeptin/Aprotinin, 5 mM NaF, 1 mM Na_3_VO_4_, 500 nM ADP-HPD, pH 7.9) on ice for 20 min with agitation. The lysates were then centrifuged at 1000 × *g* for 10 min at 4 °C and the supernatant was kept as a cytosolic fraction. The pellets were washed three times by resuspension in the cytosolic fractionation buffer and centrifugation. The resulting pellets were resuspended in high salt buffer (5 mM HEPES, 1.5 mM MgCl_2_, 0.2 mM EDTA, 0.5 mM DTT, 150 mM KCl, 300 mM NaCl, 26% glycerol (v/v), 1 mM PMSF, 10 μg/ml Leupeptin/Aprotinin, 5 mM NaF, 1 mM Na3VO4, 500 nM ADP-HPD, pH 7.9) and then centrifuged at 13,000 × *g* for 10 min at 4 °C and used as heavy membrane fraction.

### Immunoblotting

Cell lysates were prepared and boiled for 5 min at 94 °C in 2 × SDS sample buffer (0.25 M Tris-HCl pH 6.8, 2% SDS, 10% 2-mercaptoethanol, 30% glycerol, 0.01% bromophenol blue). Protein samples were separated by 8−12% SDS-polyacrylamide gel electrophoresis and immunoblotting was performed by conventional methods.

### Reverse transcription polymerase chain reaction

Total RNA was extracted using RNeasy Mini Kit (QIAGEN) according to the manufacturer`s instruction. To synthesize first strand cDNA, 1 μg of total RNA was incubated at 70 °C for 5 min with 0.5 μg of oligodT and deionized water (up to 15 μl). The reverse transcription reaction was performed using 200 units of M-MLV reverse transcriptase (Promega) in 5× reaction buffer (250 mmol/l Tris-HCl; pH 8.3, 375 mM KCl, 15 mM MgCl_2_, 50 mM DTT), 28 units of RNasin inhibitor, and 2.5 mM dNTP mixtures at 42 °C for 90 min. The primers used were; β-actin forward: 5′-TCA CCC ACA CTG TGC CCA TCT ACG AG-3, reverse: 5′-GTG GTG AAG CTG TAG CCA CGC TC-3′; PARP-1 forward: 5′-CTC GGA GAG GCT TTA TCG AGT-3′, reverse: 5′-GGA CTT GGC GTA CTC CGC TA-3′; ESP forward: 5′- GGA GCG CTC ATT TTG TCT TCC AGG TC-3′, reverse: 5′-AAG TAG TGC ACA CCA GGG TAA CCG C-3′; ASCL1 forward: 5′-ACT TGA ACT CTA TGG CGG GTT CTC CG-3′, reverse: 5′-CTT CCA AAG TCC ATT CCC AGG AGA GC-3′; neurogenin2 forward: 5′-AGA GGT GGC CCT TGC AAT CCC CTC-3′, reverse: 5′-CAC ACG CCA TAG TCC TCT TTG ACC ATA AA-3′. A final volume was 25 μl and 2 μl of the cDNA products was used to perform each PCR analysis.

### RNA microarray

Total RNA was extracted from PARP-1 littermates E13.5 NSCs using RNeasy Mini Kit (QIAGEN) according to the manufacturer`s instruction. Microarray analysis was performed using the Mouse GE 4x44K v2 Microarray Kit (Agilent) by eBiogen (commercial service). For the each RNA, the synthesis of target cRNA probes and hybridization were performed using Agilent’s LowInput QuickAmp Labeling Kit (Agilent Technologies) according to the manufacturer’s instructions. The hybridization images were analyzed by Agilent DNA microarray Scanner (Agilent Technologies) and the data quantification was performed using Agilent Feature Extraction software 10.7 (Agilent Technologies). The average fluorescence intensity for each spot was calculated and local background was subtracted. All data normalization and selection of fold-changed genes were performed using GeneSpringGX 7.3.1 (Agilent Technologies). Functional annotation of genes was performed according to Gene OntologyTM Consortium (http://www.geneontology.org/index.shtml) by GeneSpringGX 7.3.1.

### BrdU labeling

For the labeling of cultured NSCs, cells were plated onto 24 well-plate at a density of 2 × 10^4^ cells/well with fresh growth medium. Before fixation, cells were incubated in the presence of 10 μM BrdU for 15–30 min. For the in vivo labeling, BrdU (50 mg/kg) was administered intraperitoneally to adult mice two times a day for 2 consecutive days. Mice were perfused to verify stem cell proliferation 24 h after the last BrdU injection. The brain slices for BrdU staining were permeablized in 0.5% Triton X-100 for 20 min. After permeabilization, the slices were exposed to 1 N HCl at 37 °C for 30 min. The slices were then preincubated with 5% normal donkey serum. Subsequently, the slices were incubated overnight with rat anti-BrdU antibody (1:500).

### Immunostaining

Brain slices (30 μm thick) were permeabilized in 0.5% Triton X-100 for 20 min and blocked in 5% normal serum in PBS for 1 h 30 min in free floating condition. Primary antibodies were incubated for overnight in 3% normal donkey serum and 0.3% triton X-100 in PBS at 4 °C containing primary antibody. For visualization of primary antibodies, slices were incubated Alexa488 or Alexa594 anti-mouse (#A21042 or #A32742), anti-rabbit (#A11070 or #A32740), and anti-goat (#A11055 or #A11058) secondary antibodies (ThermoFisher) for 40 min. For the quantification of antibody-labeled cells in the embryonic brain sections, nonspecific signals on endothelium were excluded from the countings. Diaminobenzidine staining was used for stereological quantification of labeled cells of adult mouse brain using ABC kit according to the manufacturer’s protocol (Vector). Every seventh section (210 μm apart) were used for the counting. Stained cells were counted throughout the rostrocaudal extent of the brain (from bregma −1.58 to −2.54). Resulting numbers were multiplied by 7 to obtain the estimated total number of stained cells per a mouse.

### Nissl staining

Brain sections (10 μm thick) were mounted onto glass slides in PBS and dried overnight. For Nissl staining, slides were immersed in 0.025% cresyl violet in 90 mM acetic acid, 10 mM sodium acetate for 30 min. The stained samples were serially dehydrated in ascending concentrations of ethanol and then mounted with VectaMount permanent medium (Vector Laboratories).

### Measurement for ventricle area

Coronal cross-section images of Nissl-stained embryonic brains were used for quantification analysis using ImageJ program (US National Institute of Health). Embryonic brains were snap-frozen in OCT medium and coronal sections at the same sectional distance were collected from each brain and labeled accordingly. The matching brain sections from rostral to caudal levels were confirmed by examination under a microscope following Nissl staining. Total brain area or ventricle area was calculated by the measure function of imageJ software.

### Behavioral tests

WT and PARP-1 KO male mice (12–16 weeks) were randomly assigned to open field, elevated plus maze (EPM), social interaction, forced swim, Y-maze, novel object recognition, pre-pulse inhibition (PPI) and inverted grid tests.

#### Open Field test

Open field test was conducted in a novel open box (40 × 40 × 30 cm) under a dim light condition (40 lx). Animals were transported to the experimental room and left uninterrupted for 10 min before the experiment. Animals were placed in the center (20 × 20 cm) of a novel open field arena and both duration of time that animals spent at the center and frequency that animals visited the center were recorded. EthoVision 3.0 software (Noldus) was used to record and analyze the movement of the mice.

#### EPM test

EPM was comprised of two open arms (30 × 5 × 2 cm) and two closed arms (30 × 5 × 30 cm) that extended from center area (10 × 10 cm). The maze was elevated 50 cm above the floor. The level of anxiety was assessed by the percentage of time spent in the open arms and number of entries in the open arms.

#### Social interaction test

The social interaction test was conducted in the same arena used for the open field test box. A cylinder was placed at the center of the open box. Animals were placed around the cylinder and were allowed to explore for 5 min. Next day, the mice were relocated to the around of the cylinder in which a novel stranger (Non-experiment mice) was placed. The time that animals were approaching, sniffing, kicking and staring were recorded.

#### Forced swim test

Animals were put in an open glass cylinder (diameter, 15 cm; height, 25 cm) filled with room temperature water to a depth of 15 cm. The immobility times were measured during a 5-min test. A mouse was considered to be immobile when it ceased struggling to escape and remained floating motionless in the water. Water was replaced between every trial.

#### Y-maze test

The Y-maze is composed of three identical arms placed at 120 degrees with respect to each other. Each arm was 40 cm long and 12 cm height, 3 cm wide at the bottom and 10 cm wide at the top and converged at an equilateral triangle center area. Each mouse positioned at end of the arm and allowed to explore the Y-maze freely for 12 min. An alternation was defined as the entry into all three arms on consecutive choice. Percent alternations were calculated using the formula: 100 × Total number of alternations/(Total number of arm entries – 2).

#### Novel object recognition test

The Novel object recognition training took place in the same arena used for the open field test. The training was conducted by placing an individual animal in the box, in which objects A and B were positioned in two adjacent corner 10 cm from the walls. The animal was left to explore the objects for 5 min. The animal was returned 24 h after training to the box where one of the original objects has been replaced by a new object C and allowed to explore the objects freely for 5 min. The amount of time that the animals spent exploring each object was measured. A recognition index calculated for each animal was expressed by the ratio TC/(TB + TC) (TB = time spent exploring the familiar object B, TC = time spent exploring the novel object C).

#### PPI test

PPI of acoustic startle response was measured with SR-LAB startle chambers (San Diego Instruments, San Diego, CA). An animal was allowed 15-min habituation to the apparatus with a continuous background noise of 70 dB and then exposed to the various acoustic stimuli. All PPI test sessions consisted of startle and prepulse trials. The startle trial consisted of a 40-ms 120-dB startle pulse of broad-band noise. Prepulse trials consisted of a 20-ms noise prepulse, 80-ms delay, and then a 40-ms 120-dB startle pulse. The prepulse intensities were 72, 74, 76, and 80 dB (i.e., 2, 4, 6, and 10 dB above the 70-dB background noise). A test session consisted of six trial types (i.e., two types for startle trial and in between four types for prepulse trials were presented in a pseudo-random order and the average interval between the trials was 15 s (range: 12–30 s). A background noise level of 70 dB was presented throughout the test session. The startle response was recorded for 65 ms at 1-ms intervals starting from the startle stimulus onset, and the maximum startle amplitude was used as the dependent variable. The PPI for each prepulse trial was defined as 100 - {[(startle amplitude for prepulse trials)/(startle amplitude for startle trials)] × 100}.

#### Inverted grid test

Mice were placed on 1 cm^2^-spaced metal grid and then the grid was slowly inverted 35 cm above the ground covered with soft bedding. Then the time for the mouse to fall onto the ground was measured within 4 min. Mice were given two times of practice 5 min before the test. Each mouse was tested three times with a rest interval of 2 min between each trial and falls within 10 s were not included^46^.

### Statistics

For the statistical analysis of in vitro data, all the experiments were repeated at least three times unless stated otherwise and the results were expressed as mean ± SD. The results of behavioral tests were expressed as mean ± SEM. No statistical methods were applied to predetermine sample sizes. However, our sample sizes are similar to those reported in previous studies for the cell samples and animal numbers. Experimenters were not blinded during data acquisition, but analyses were performed blind to genotype and experimental group. No data were excluded from the study. Data were compared using two-tailed unpaired *t*-test, Mann–Whitney test, Kruskal–Wallis test, one-way ANOVA and two-way repeated measures ANOVA, where appropriate. Normality of data distribution was tested by D’Agostino and Pearson omnibus normality test. Normal distributions were compared using two-tailed unpaired *t*-test and ANOVAs with Bonferroni post hoc comparison. Non-normal distributions were compared using Mann–Whitney test and Kruskal–Wallis test with Dunn’s post hoc comparisons. All data were plotted and analyzed using Prism 6.0 (GraphPad Software). Statistical significance was defined at level of *p* < 0.05.

## Supplementary information

Supplementary figure legends

Supplementary figure 1

Supplementary figure 2

Supplementary figure 3

Supplementary figure 4

Supplementary figure 5

Supplementary figure 6

Supplementary figure 7
