# Supplementary material for: Defective neurogenesis and schizophrenia-like behavior in PARP-1-deficient mice
Source: Cell Death Dis. 2019 Dec 9;10(12):943. doi: 10.1038/s41419-019-2174-0 (PMC6901579; doi:10.1038/s41419-019-2174-0)
Supplement: Supplementary file 1 — Supplementary figure legends [file 41419_2019_2174_MOESM1_ESM.docx]

**Supplementary figure legends**

**Fig S1**. **A**. Hierachical clustering based on gene ontology of wild-type vs. PARP-1 knockout NSCs. Genes with more than 2-fold changes were included. **B**. Fold changes and raw values of receptor-type protein tyrosine phosphatase members are listed.

**Fig S2**. Expression patterns of neuronal and glial markers in the cortex of E17 embryos. Brain sections were immunostained with antibodies of the indicated markers of brain development. **A**. Nestin-immunoreactive cells in the cortex of WT and PARP-1 KO brains. No noticeable changes were observed. **B**. Tbr2-immunoreactive cells in the cortex of WT and PARP-1 KO brains. The number of Tbr2-positive cells was greater in WT than that of PARP-1 KO (*p < 0.05, two-tailed unpaired t-test, n = 4 animals for each, mean ± SEM). **C**. Olig2-immunoreactive cells in the cortex of WT and KO brains. The number of Olig2-positive cells was increased in the PARP-1 KO (*p < 0.05, two-tailed unpaired *t*-test, n = 4 animals for each group, mean ± SEM). Counting area in B and C was set to include 100 ~ 200 cells per area and equally applied for comparable location in each section. D. Brain cortices from E16.5 embryos of PARP-1 WT and KO (3 embryos each) were processed for immunoblot analysis to monitor the protein expression levels of nestin, Tbr2, and Olig2. The band intensities were quantified using ImageJ (bottom panels; *p < 0.05, two-tailed unpaired *t*-test, n = 3 animals).

**Fig S3**. NeuN- and GFAP-positive cells were examined in the adult brains of WT and PARP-1 KO mice (12-16 weeks old). Overall, no remarkable difference in the hippocampus and the somatosensory cortex was observed beween WT and PARP-1 KO mice. **A**. Representative fluorescent images of NeuN-immunostained cells in the hippocampus. **B**. Thickness of the layers from four comparable sections were measured and averaged to represent each as mean ± SEM. There was no significant difference between WT and PARP-1 KO (CA1: CA1 pyramidal cell layer, p = 0.8435; upper blade: upper blade of the dentate gyrus, p = 0.5650; lower blade: lower blade of the dentate gyrus, p = 0.3416; two-tailed unpaired t-test, n = 4 for each group). **C**. Representative fluorescent images of NeuN-immunostained cells in the somatosensory cortex. **D**. NeuN-positive neurons in the somatosensory cortex were quantified. NeuN-positive neurons from comparable sections were counted and averaged to represent each as mean ± SEM. There was a tendency of increase in­ NeuN-positive neurons of WT compared to PARP-1 KO, but it did not reach statistical significance (p = 0.0907, two-tailed unpaired t-test, n = 4 for each group). **E**. The protein levels of NeuN was examined by immunoblot analysis using cortices from WT and PARP-1 KO adult mice (n = 3 each). The band intensities were quantified using imageJ (shown in the right panel; *p < 0.05, two-tailed unpaired *t*-test, n = 3 animals). Note that no significant changes were detected. **F.** Representative fluorescent images of GFAP-immunostained cells in the hippocampus. **G**. The cumulative distribution plot for GFAP fluorescence intensity in the hippocampus calculated from 6 comparable sections shows no significant shift between the groups (D = 0.096, p = 0.1740, Kolmogorov-Simimov test). **H**. Representative fluorescent images of GFAP-positive cells in the somatosensory cortex. **I**. GFAP-positive cells in the somatosensory cortex from comparable sections were counted and averaged to represent each as mean ± SEM. No significant difference was found between the groups (p = 0.8094, two-tailed unpaired t-test, n = 4 animals each).

**Fig S4**. PARP-1 deficiency affects NSC proliferation in the adult DG. Brain sections were stained with anti-Ki67 and anti-SOX2 antibodies and quantitative analysis of Ki67- and SOX2-positive cells was performed. SOX2- (**A**) and Ki67-positive cells (**B**) were decreased in the DG of PARP-1 KO mice when compared with WT. Scale bar = 50 μm. *p < 0.05, two-tailed unpaired t-test, n = 4 for each group, mean ± SEM.

**Fig S5**. PARP-1 deficiency affects neuronal differentiation of progenitors in the adult DG. Adult WT and PARP-1 KO brains were immunostained for NeuroD (**A**) and DCX (**B**) and the positive cells were counted in the DG area. The numbers of NeuroD1- and DCX-positive cells were significantly smaller in PARP-1 KO mice compared with those in WT mice. Scale bar = 50 μm. *p < 0.05, ***p < 0.001, two-tailed unpaired t-test, n = 4 for NeuroD1 and n = 3–4 for DCX, mean ± SEM.

**Fig S6.** Reversal of PPI deficit in PARP-1 KO mice by haloperidol. Haloperidol (0.2 mg/kg intraperitoneal injection) was administered 30 min before the test. The PPI deficit observed in PARP-1 KO was restored by haloperidol. Two-way repeated measures ANOVA detected no significant effect of genotype (F_1,6_ = 0.14, P = 0.9094) and interaction between genotype and prepulse intensity (F_3,18_ = 1.307, P = 0.3027). n = 4 for each, mean ± SEM.

**Fig S7**. Normal moving velocity and muscle strength in PARP-1 KO mice. **A.** Upper panel shows representative 10 min trajectories of WT and KO mice in open field. Measured average velocity of movement in open filed is shown in lower panel. There were no differences between WT and KO mice in overall activity in the open-field test (ns: not significant, p = 0.5047, two-tailed unpaired t-test, n = 46 animals for each group). **B.** Muscle functionality as a sign of general health was assessed by inverted grid test. The latency to fall off the upside-down grid was quantified; a 240-sec cutoff time was used. No significant differences were found between WT and PAPR-1 KO aged 12-16 weeks. (ns: not significant, p = 0.9198, two-tailed unpaired t-test, n = 4 animals for each, mean ± SEM).
