# Supplementary figures and images for: Defective neurogenesis and schizophrenia-like behavior in PARP-1-deficient mice
Source: Cell Death Dis. 2019 Dec 9;10(12):943. doi: 10.1038/s41419-019-2174-0 (PMC6901579; doi:10.1038/s41419-019-2174-0)

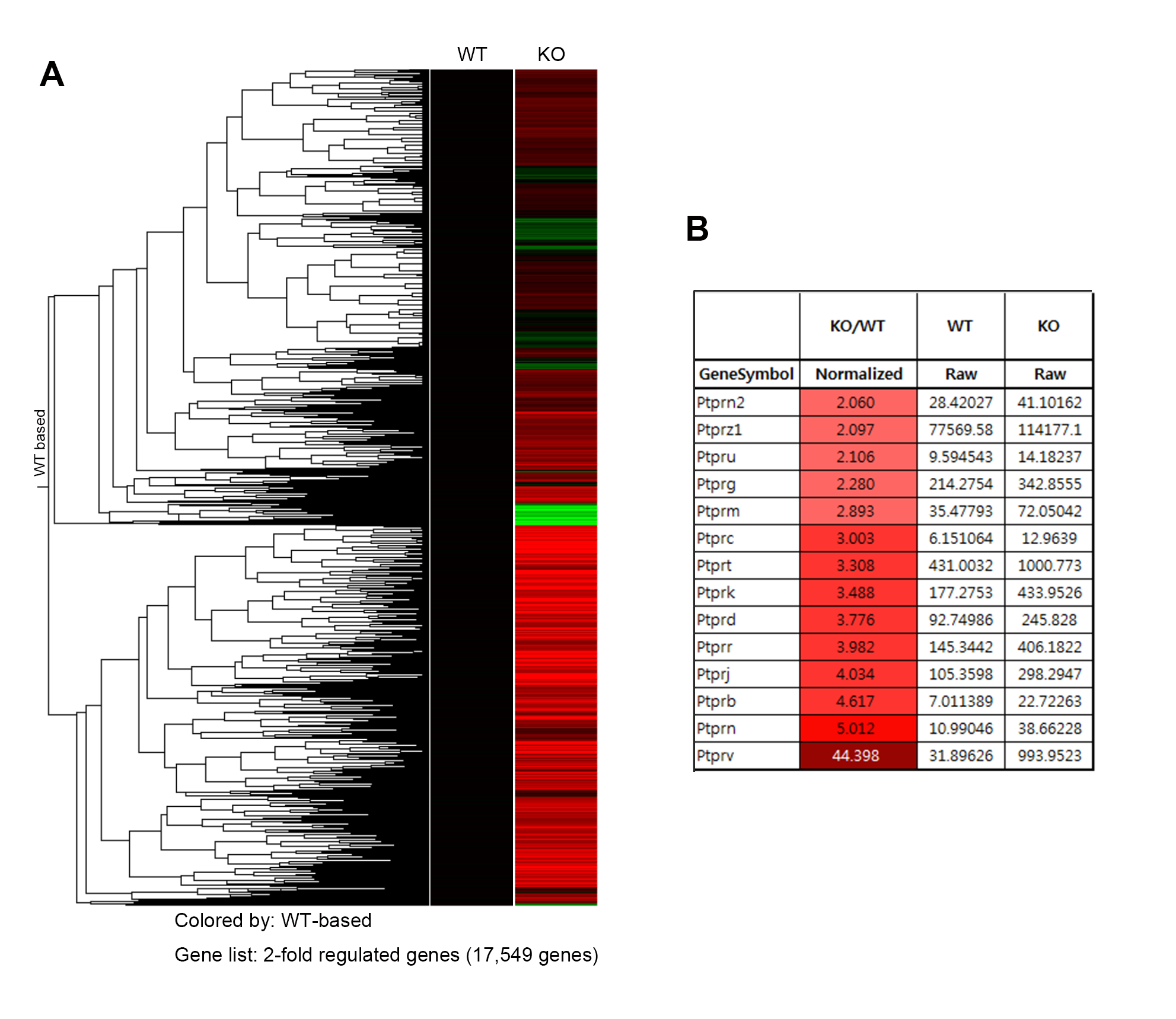

Supplement: Supplementary file 2 — Supplementary figure 1 [file 41419_2019_2174_MOESM2_ESM.tif]

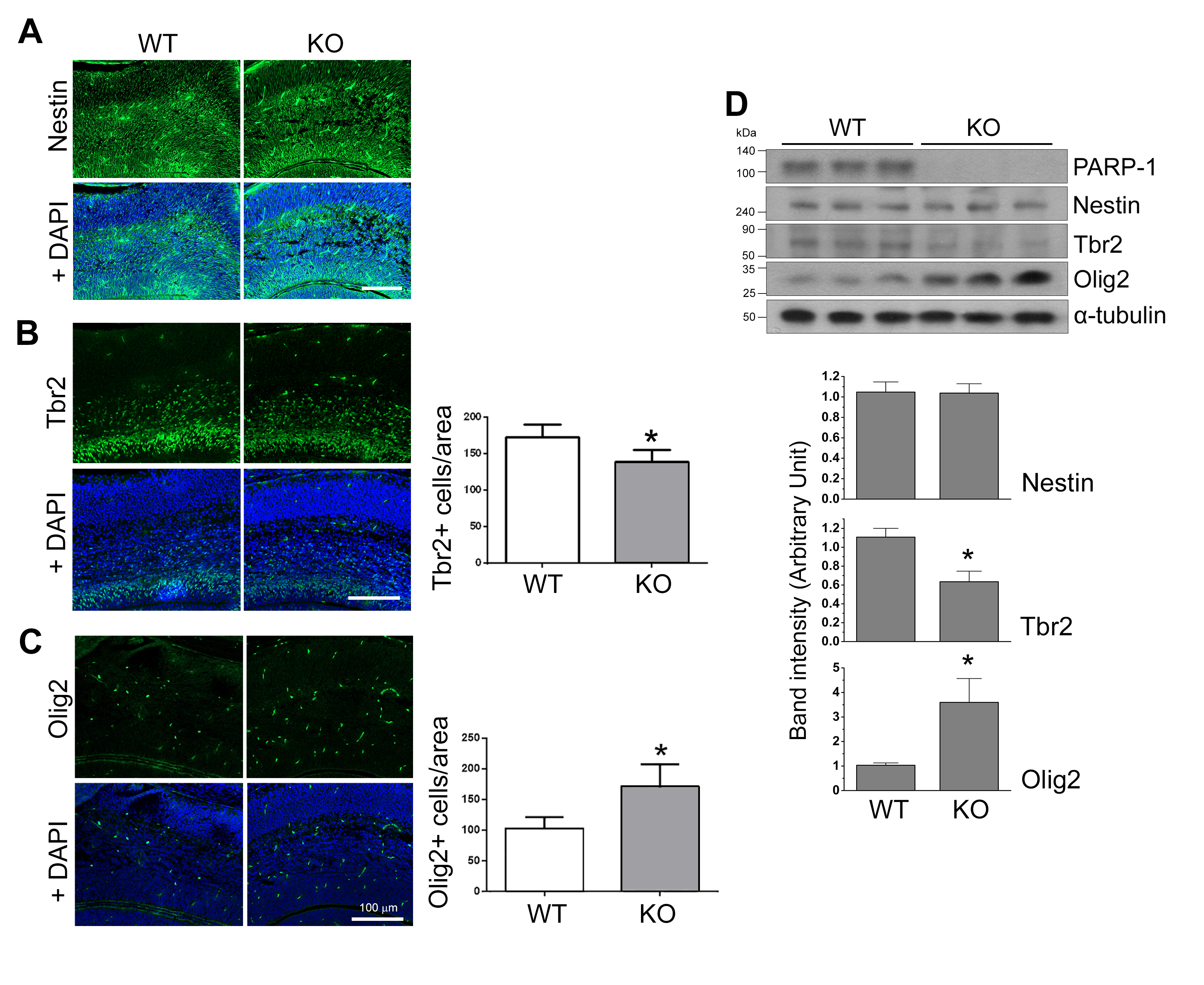

Supplement: Supplementary file 3 — Supplementary figure 2 [file 41419_2019_2174_MOESM3_ESM.tif]

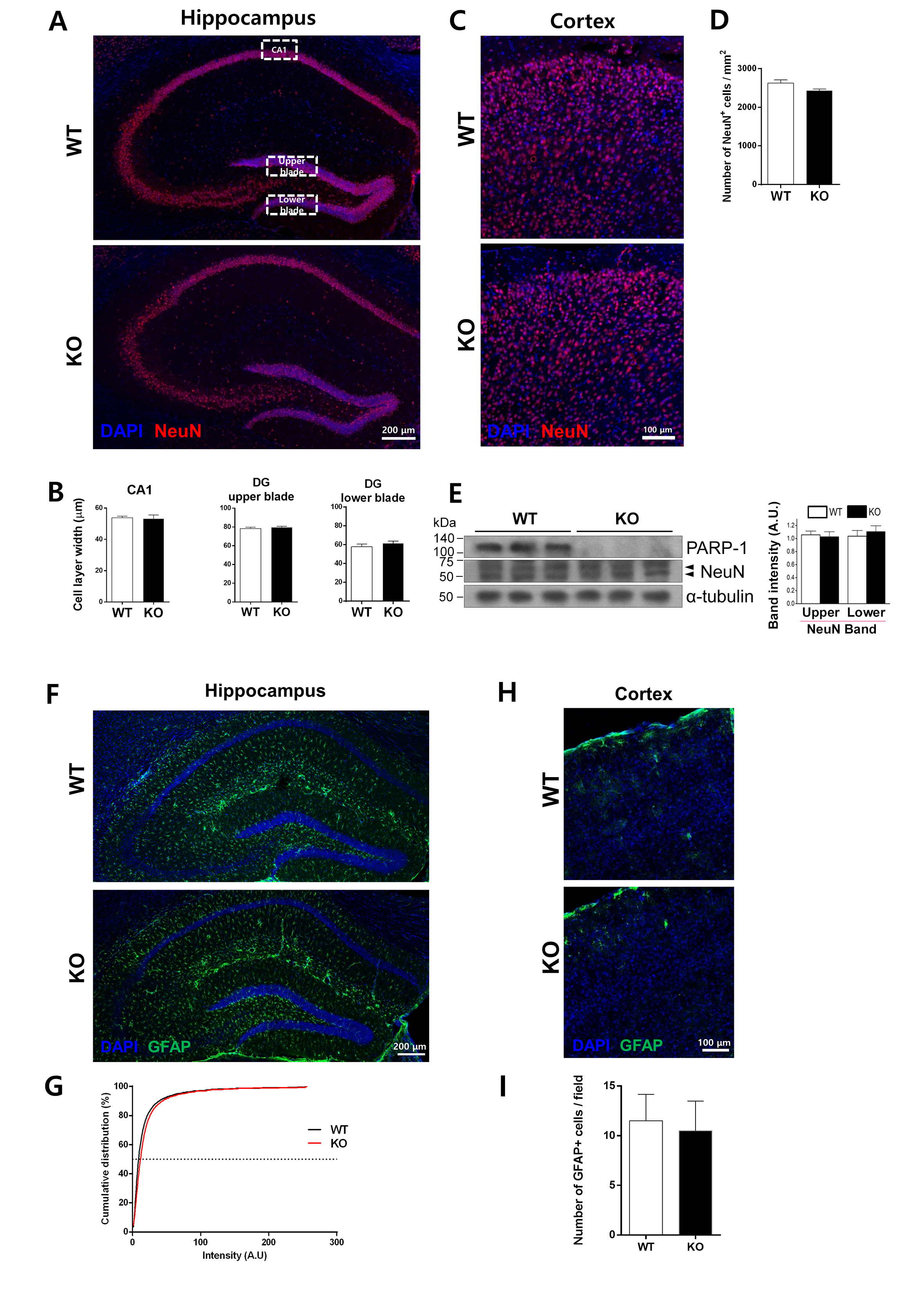

Supplement: Supplementary file 4 — Supplementary figure 3 [file 41419_2019_2174_MOESM4_ESM.tif]

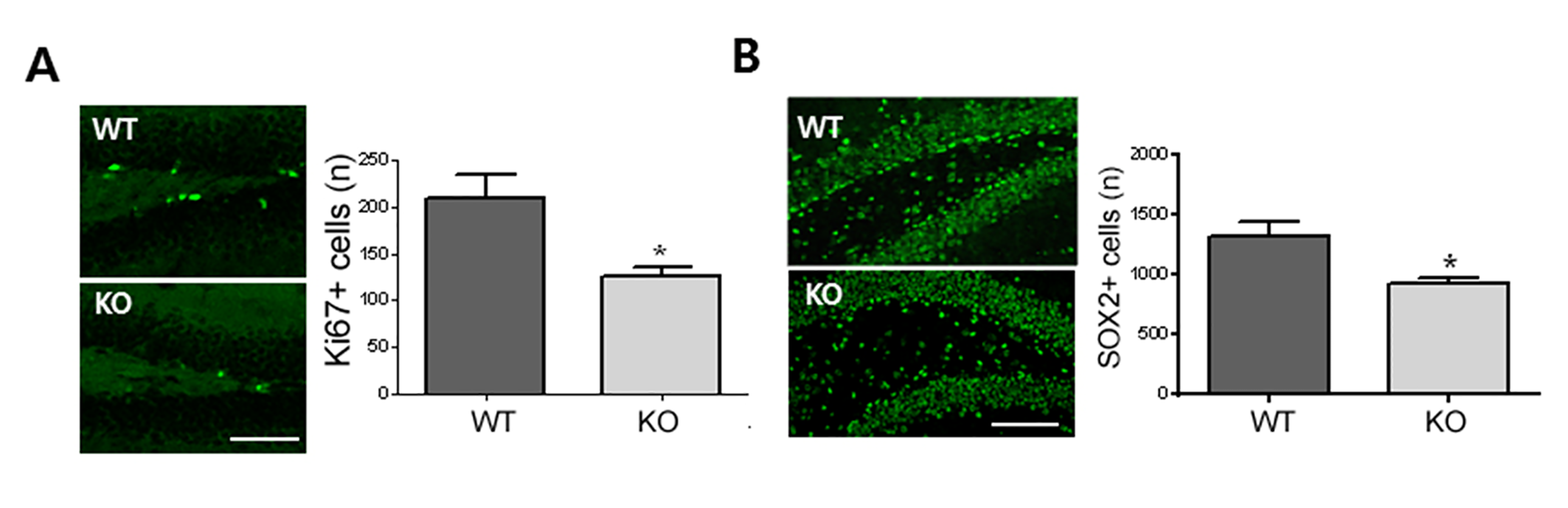

Supplement: Supplementary file 5 — Supplementary figure 4 [file 41419_2019_2174_MOESM5_ESM.tif]

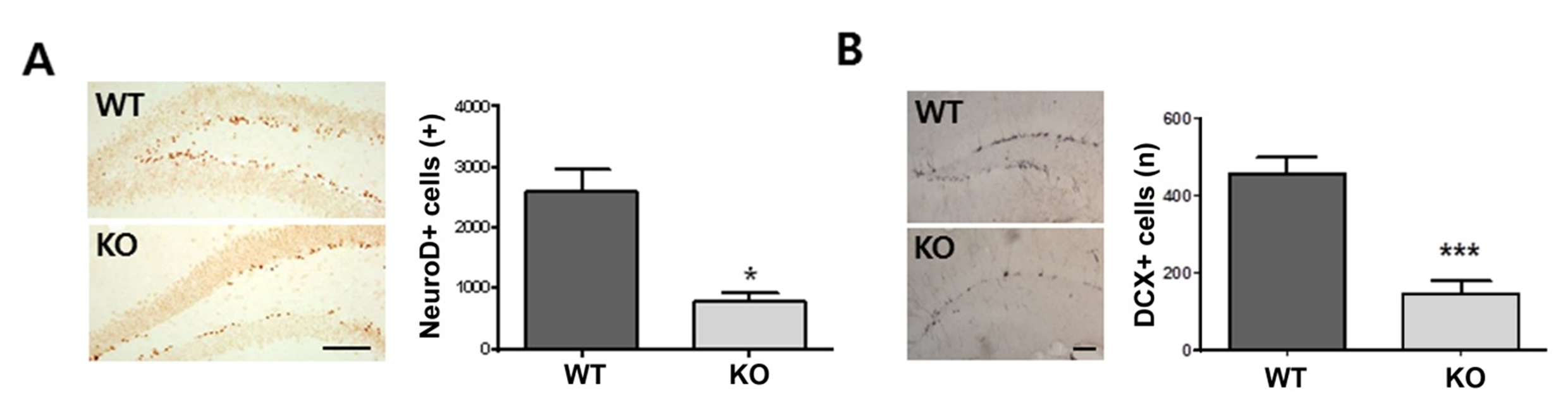

Supplement: Supplementary file 6 — Supplementary figure 5 [file 41419_2019_2174_MOESM6_ESM.tif]

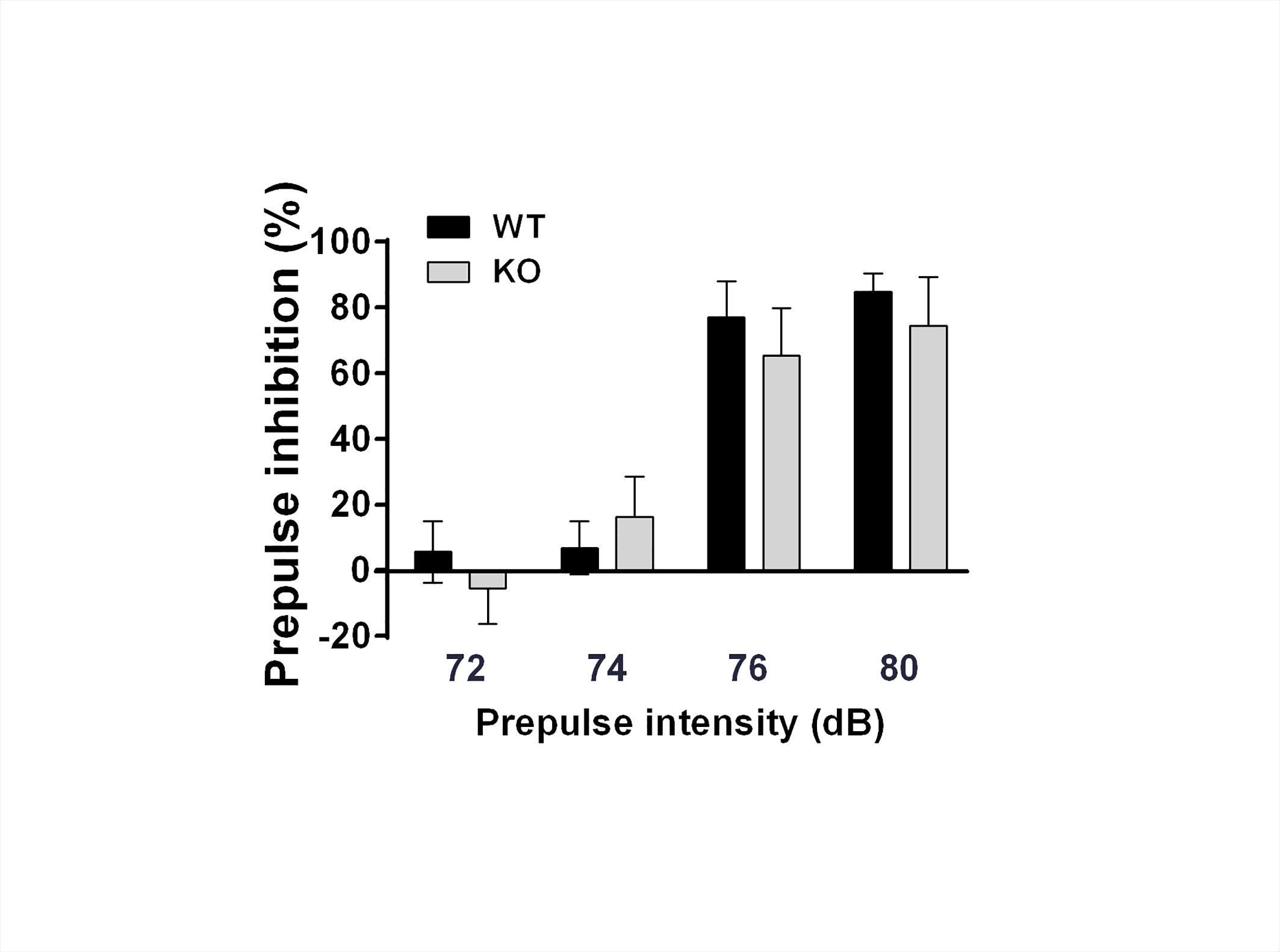

Supplement: Supplementary file 7 — Supplementary figure 6 [file 41419_2019_2174_MOESM7_ESM.tif]

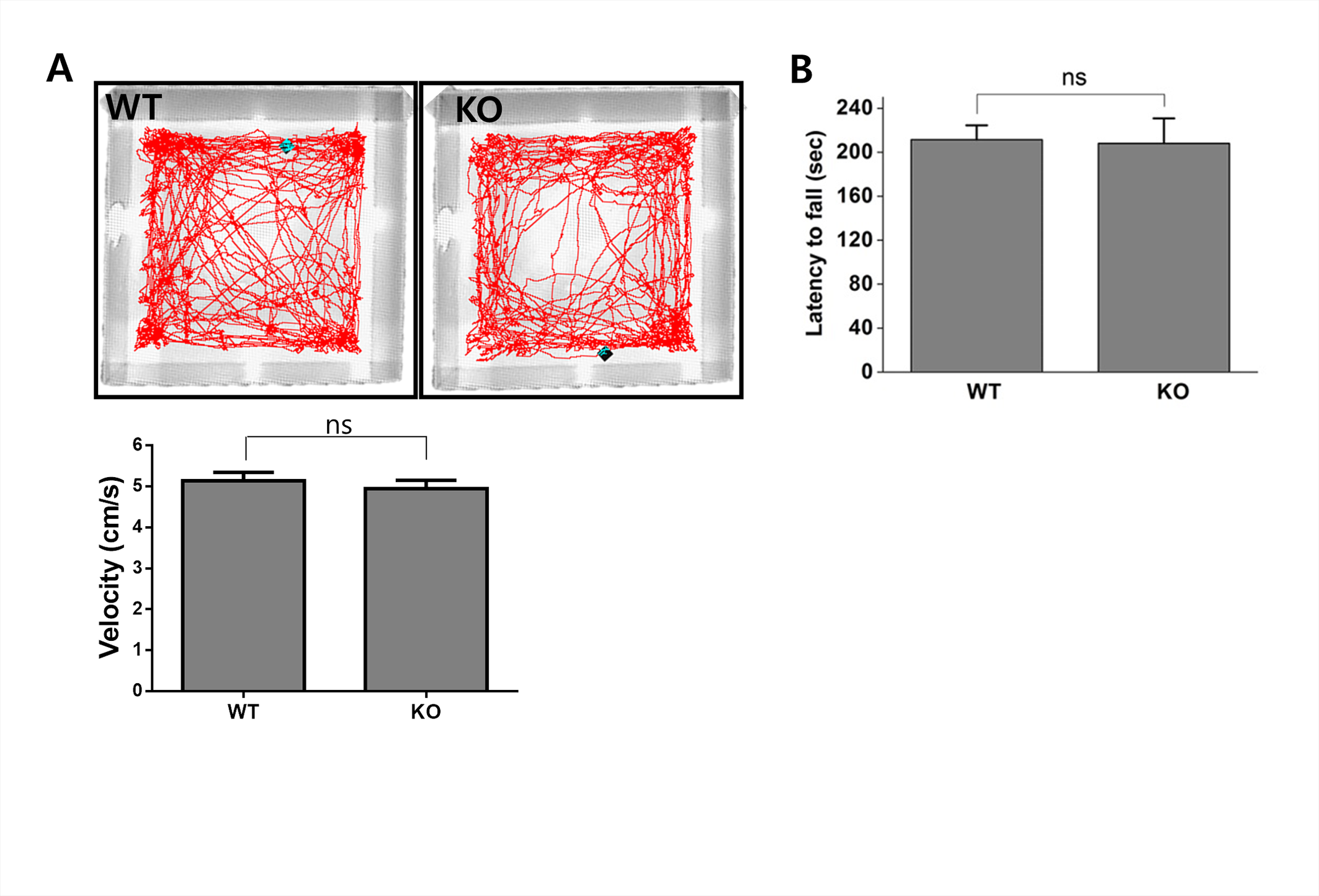

Supplement: Supplementary file 8 — Supplementary figure 7 [file 41419_2019_2174_MOESM8_ESM.tif]
